# Comparative transcriptomic analysis and structure prediction of novel Newt proteins

**DOI:** 10.1371/journal.pone.0220416

**Published:** 2019-08-16

**Authors:** Abijeet Singh Mehta, Agustin Luz-Madrigal, Jian-Liang Li, Panagiotis A. Tsonis, Amit Singh

**Affiliations:** 1 Department of Biology, University of Dayton, Dayton, Ohio, United States of America; 2 Sanford Burnham Prebys Medical Discovery Institute at Lake Nona, Orlando, Florida, United States of America; 3 Premedical Program, University of Dayton, Dayton, Ohio, United States of America; 4 Center for Tissue Regeneration and Engineering at Dayton (TREND), University of Dayton, Dayton, Ohio, United States of America; 5 The Integrative Science and Engineering Center, University of Dayton, Dayton, Ohio, United States of America; 6 Center for Genomic Advocacy (TCGA), Indiana State University, Terre Haute, Indiana, United States of America; National Cancer Institute, UNITED STATES

## Abstract

*Notophthalmus viridescens* (Red-spotted Newt) possess amazing capabilities to regenerate their organs and other tissues. Previously, using a *de novo* assembly of the newt transcriptome combined with proteomic validation, our group identified a novel family of five protein members expressed in adult tissues during regeneration in *Notophthalmus viridescens*. The presence of a putative signal peptide suggests that all these proteins are secretory in nature. Here we employed iterative threading assembly refinement (I-TASSER) server to generate three-dimensional structure of these novel Newt proteins and predicted their function. Our data suggests that these proteins could act as ion transporters, and be involved in redox reaction(s). Due to absence of transgenic approaches in *N*. *viridescens*, and conservation of genetic machinery across species, we generated transgenic *Drosophila melanogaster* to misexpress these genes. Expression of 2775 transcripts were compared between these five newly identified Newt genes. We found that genes involved in the developmental process, cell cycle, apoptosis, and immune response are among those that are highly enriched. To validate the RNA Seq. data, expression of six highly regulated genes were verified using real time Quantitative Polymerase Chain Reaction (RT-qPCR). These graded gene expression patterns provide insight into the function of novel protein family identified in Newt, and layout a map for future studies in the field.

## Introduction

Urodeles (salamanders and newts) have evolved during the Permian period, the last period of the Paleozoic era, ∼300 million years ago [1]. Newts are among the few vertebrates that possess amazing capability to regenerate tissues like limbs, tail, heart, lens, spinal cord, brain, retina etc. [2]. Many evolutionary conserved pathways, like Wnt [3], Hedgehog (Hh) [4], Notch (N) [5], and Bone morphogenetic protein (BMP) [6] etc., have been reported to play role during Newt regeneration. Similar pathways are known to promote cell proliferation during regeneration of mammalian muscle, liver and bone [7]. Newts do not lose the capacity to regenerate even after repetitive tissue damage that continues over several years [8, 9]. However, mammalians (homeothermic vertebrate) have a limited regeneration potential that declines rapidly during postnatal life, and paradoxically the risk of cancer increases [10]. Therefore, it raises the speculation that regulating such conserved pathways, like Wnt, Hh, N, BMP etc. could promote tissue regeneration in mammals [11].

Previously our group using *de novo* assembly of a comprehensive collection of tissue-specific transcripts of *Notophthalmus viridescens* combined with proteomic validation identified a novel family of five protein members expressed in the adult tissues and during regeneration [12]. The presence of a putative signal peptide suggests that all these proteins may be secreted. We used I-TASSER server to generate three-dimensional structure of Newt candidate proteins and predicted their function [13]. Since there are challenges in generating transgenic *Notophthalmus viridescens*, we used genetically tractable model of *Drosophila melanogaster* to look into the biological function of these Newt-specific genes. The rationale was that these signaling pathways, which are involved in regeneration, are evolutionarily conserved across the species [11].

*Drosophila melanogaster*, also called as fruit fly, is the member of super phylum Ecdysozoa [14]. *Drosophila* is one of the highly versatile genetic models available to the scientific community [15, 16]. *Drosophila* has a short life cycle of 12 days [15], and a large repository of mutants and transgenic animals are readily available [17]. Moreover, the genetic machinery is highly conserved across the species. Therefore, the results generated from studies in flies can be extrapolated to humans and other vertebrates. This makes *Drosophila* a suitable animal model for cross species studies where we can ascertain the mechanism behind the function of genes from animals that have limited genetic tools, and have longer life cycle e.g. Newts, Mammals etc. [18–20]. Here, we employed next generation RNA-sequencing to report for the first time the expression of 2775 annotated transcripts that have been differentially regulated (significant) when newly identified Newt genes were misexpressed in *Drosophila*.

## Materials and methods

### Animals

Handling of *Notophthalmus viridescens* have been described previously [21]. Briefly, Newts were anesthetized in 0.1% (w/v) ethyl-3-aminobenzoate methanesulfonic acid (MS222; Sigma) in phosphate buffered saline. Surgery was performed using a scalpel to cut the tail. All procedures involving animals were approved by the University of Dayton Institutional Animal Care and Use Committee (IACUC; Protocol ID: 011–02). All surgical procedures were performed in Newts anesthetized with MS222. All appropriate procedures were used to alleviate pain and distress to animals.

### Protein structure and function predictions

Using I-TASSER server https://zhanglab.ccmb.med.umich.edu/I-TASSER/ three-dimensional structure of Newt genes were constructed and their function: structural similarity, and binding partners were predicted [13]. Briefly, server first retrieve structural templates of similar folds from the protein database (PDB) by locally installed meta-threading approach (LOMETS), followed with full-length atomic models constructed by iterative template-based fragment assembly simulations. Functional insights of the target were derived by threading the 3D models through protein function database BioLip.

### Sample preparation to clone Newt candidate genes

Total RNA was extracted from the newt tail using Nucleospin RNA II isolation kit (Macherey-Nagel, Germany) following the manufacture’s protocol. The quality and quantity of RNA was determined using Agilent RNA 6000 nano LabChip (Agilent 2100 Bioanalyzer). Approximately 200ng of total RNA with a RIN >9 were used for the cDNA synthesis using ImProm-II Reverse Transcription System (Promega) and random-primer hexamers. All PCR reactions were performed using PlatinumTaq DNA polymerase (Invitrogen). The primers used are:

C1Fw 5’-AAAGGATCCatgaagatctctctagctttcc-3’,

C1Rev 5’-AAATCTAGActaagaacagctgcgacaagtg-3’

C2Fw 5’-AAAGGATCCatgaagatctctctagctttcc-3’

C2Rev 5’-AAATCTAGActaagaacagctgcgataagtgg-3’

C3Fw 5’-AAAGGATCCatgaagatctctctagctttcc-3’

C3Rev 5’-AAATCTAGActagtcgactagaaggcctgc-3’

C4Fw 5’-AAACTCGAGatgaagattgctctcgttttcc-3’

C4Rev 5’-AAATCTAGAttagcacctaccgccaggcag-3’

C5Fw 5’-AAACTCGAGatgaagatcgctctcgttttc-3’

C5Rev 5’-AAATCTAGActactgcttccacacttgccaaa-3’

The underlined sequences introduced XbaI and BamHI sites at the ends of C1, C2 and C3 and XbaI and XhoI at the ends of C4 and C5 to facilitate the cloning in pUAST-attB plasmid. The fragments were first cloned in pDrive (QIAGEN) and the sequence for each gene was confirmed using the primer M13 forward -21 (5'-GTAAAACGACGGCCAGT-3'). Thereafter, the fragments C1 (498bp), C2 (402bp) or C3 (426pb) were delivered from pDrive using the enzymes XbaI/BamHI and cloned into the sites XbaI/BglII downstream of 5XUAS-hsp70 sequence in pUAST-attB plasmid. The fragments C4 (501bp) and C5 (426bp) were delivered from pDrive using the enzymes XhoI/XbaI and cloned into the same sites in pUAST-attB plasmid.

### Generating transgenic flies

Transgenic flies were generated using microinjection-based ϕC31 integrase mRNA-mediated method. A cloned candidate gene using pUAST-attB plasmid containing both a transgene and donor sequence (attB) is coinjected along with ϕC31 integrase mRNA into attP-containing recipient embryos, resulting in the site-specific insertion of the transgene [22, 23]. Following this procedure, five independent transgenic flies were generated with insertion at Chromosome III. Flies were genotyped to verify Newt gene insertion in *Drosophila* genome. Targeted misexpression of Newt genes in *Drosophila* was achieved using Gal4/UAS binary system [24].

**RNA sequencing** (Protocol.io dx.doi.org/10.17504/protocols.io.5bng2me).

Illumina reads were mapped to the *Drosophila* genome dm6 using TopHat splice-aware aligner [25]. Expectation-Maximization (EM) approach was used to estimate transcript abundance [26]. Reads per kilobase per million mapped reads (RPKM) approach was applied for within sample normalization [27]. Between sample normalization and differentially expressed test were performed by BioConductor DESeq package (v 1.20), which allows analysis of non-replicate experiments [28]. The significant criteria were (1) detected transcript in at least one sample (RPKM>1), (2) fold change over 2 and (3) adjusted p-value less than 0.05 [29].

Enriched gene ontology (GO) terms were identified using gene ontology enrichment analysis and visualization tool (GOrilla) [30]. Single ranked list of genes was chosen as a running mode criterion, and for enriched GO terms, searched P-value threshold was equal to 10^−3^. Using reduce + visualize gene ontology (REVIGO) tool long lists of gene ontology terms obtained above (by running GOrilla) are summarized by removing redundant GO terms [31]. The highly enriched terms are visualized as bar graph. We also used protein analysis through evolutionary relationships (PANTHER) 14.1 version as a tool to select set of enriched gene ontology terms for classifications by molecular function, cellular component, and Protein class [32].

### Real time Quantitative Polymerase Chain Reaction (RT-qPCR)

Collected tissue was homogenized and total RNA was extracted following TRIzol Reagent protocol (Applied Biosciences). Aqueous phase was transferred to RNA Clean & Concentrator (Zymo research, Cat. No. R1080) columns. Quality of isolated RNA was determined by Nanodrop 2000 spectrophotometer (Thermo Scientific). cDNA was produced from total RNA through Reverse transcription reaction (RT) using first- strand cDNA synthesis kit (GE healthcare, Cat# 27926101). RT-qPCR was performed according to the standard protocol [33]. The primers used are:

GAPDHFw5’-CCGTTGACCACCAGGAAA-3’

GAPDHRev5’-CAATGGATTTGGTCGCATCG-3’

pka-C1Fw5’-CGTATTCGCCTCCCTGTTATT

pka-C1Rev5’-CGAGTTGCTCTCGCTCTTTAT

Hsp 70BbFw5’-GATGGTGCTGACCAAGATGAA

Hsp 70BbRev5’-CGCTGAGAGTCGTTGAAGTAAG

PGRP-SB2Fw5’-CAGTTGGCTCTCGTTCTATGT

PGRP-SB2Rev5’-ATGAGCCTGGGCATTCG

CG12224Fw5’-TGGACAGGGTGGACATACTA

CG12224Rev5’- GACGGGTATGGTCTCATTCAG

SypFw5’-AAGTGCAGAAGGATGAAGGG

SypRev5’-TTTGCTGCAACTGGGAATTG

Unc-115bFw5’-CAGACAAGACCAGACCGATATAC

Unc-115bRev5’-GTGCATTTCGCACAGTAGATTT

### Statistical analysis for RT-qPCR results

Statistical analysis was performed using two-way analysis of variance (ANOVA) and Student’s t-test for independent samples. Samples were run in triplicates (n = 3). Statistical significance was determined with 95% confidence (p<0.05). Equal variances for student’s t-test were assumed when Levene’s test p value was greater than 0.05.

## Results

### Protein structure and function prediction

**Fig 1:**
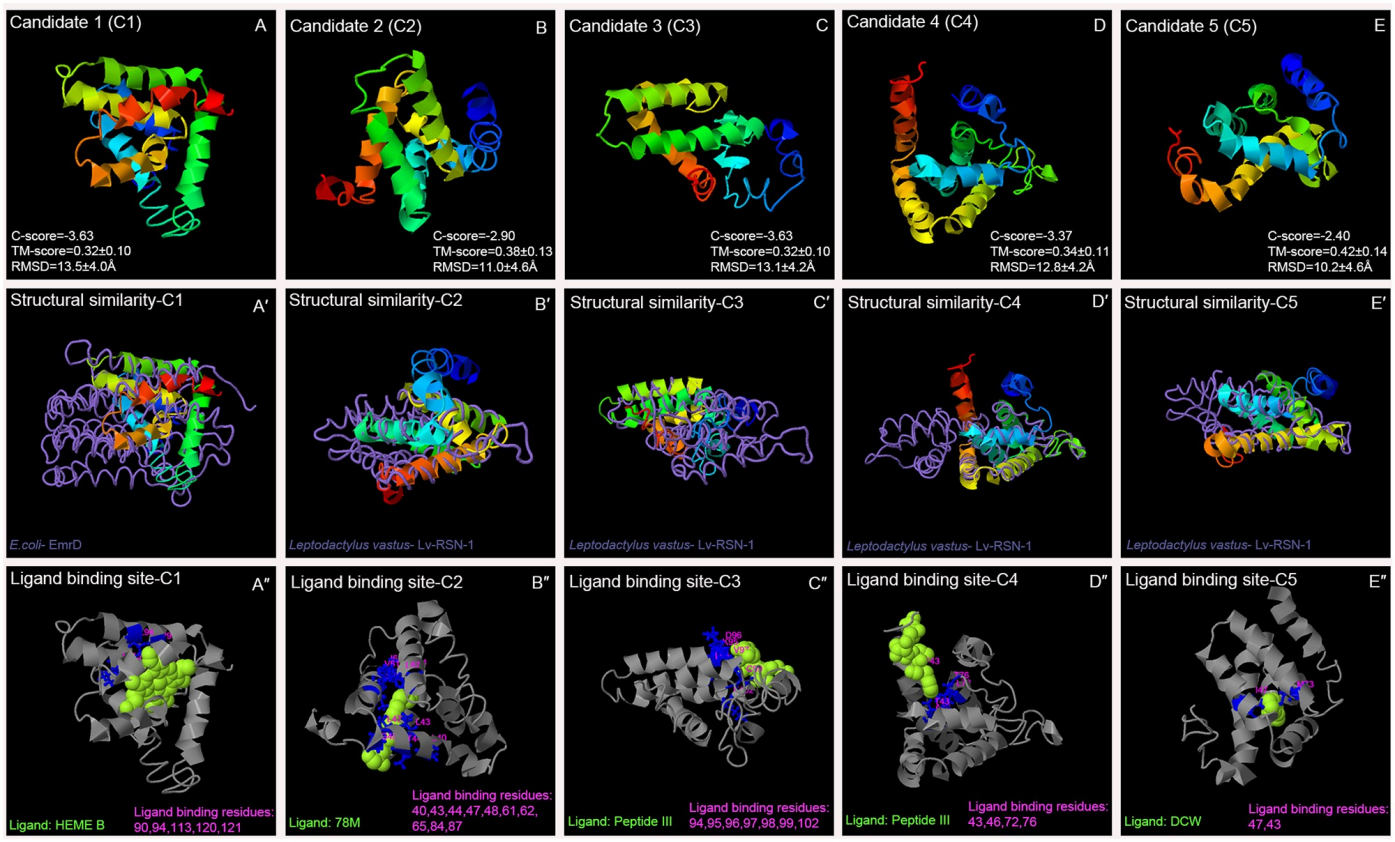
Three-dimensional structure of novel Newt proteins and their predicted function. Using I-TASSER server (A-E) Three-dimensional structure of novel Newt proteins (A'-E') Structural similarity to already submitted proteins from PDB library (Query structure is shown in the cartoon, while the structural analog is displayed using backbone trace), and (A''-E'') ligand that has highest probability to bind to the ligand site in the protein are predicted.

I-TASSER server reported five models for each protein, which corresponds to the five largest structure clusters. The confidence of each model is quantitatively measured by C-score, which is calculated based on the significance of threading template alignments and the convergence parameters of the structure assembly simulations [13]. Out of the five models, the one with higher C- score is selected as the best-fit protein model for a respective Newt protein (Fig 1A–1E).

Best-fit model for all five Newt proteins were found to have C-score values greater than -4. C-score is typically in the range of [–5, 2], where a C-score of a higher value signifies a model with a higher confidence and *vice-versa*. Residue number of the protein models and its corresponding (1) predicted secondary structure (SS): C—random coil; H—alpha-helix; S—beta-strand, (2) predicted solvent accessibility (SA) at 25% cutoff: E—exposed; B–buried, (3) threading alignment coverage (COV) (4) predicted normalized B-factor (BFP), and (5) Residue-Specific Quality (RSQ) are shown as (S1 Table). RSQ, measured as the local accuracy, is defined as the estimated deviation of the residue on the model from the native structure of the protein. Since the native structure is unknown, the distance errors in the following plots are estimated by ResQ [34]. Average distance error for our results is approximately ± 2. Large-scale benchmark tests show that the estimated local accuracy has an average distance error of < 2.21 Angstrom [34]. Our results demonstrate that approximately 70% of the residue of all 5 predicted Newt proteins are accessible to solvent. Most of these residue form alpha helix, followed by random coil, and least form the beta strands. More than 70% of the residue’s BFP values are less than zero. BFP value for models higher than zero are less stable in experimental structures [34].

To predict function, I-TASSER used the TM-align structural alignment program to match the best-fit I-TASSER model to all structures in the PDB library. Top 10 proteins generated from the PDB have closest structural similarity, which is based on the highest TM-score to the predicted I-TASSER model (S2 Table). The best-fit structural similarity to the corresponding Newt candidate protein is shown in (Fig 1A'–1E'). We found that Newt proteins show similarity to (1) transport proteins *e*.*g*., EmrD, H+/Ca2+ exchanger CAX, photosynthetic reaction center, Sodium/Sugar symporter, carnitine transporter, cationic amino acid transporter etc. (2) Proteins involved in redox reaction *e*.*g*. Nitric oxide reductase, Recombinant Cytochrome ba_3_ Oxidase, 1,2-propanediol oxidoreductase etc. (3) membrane bound proteins e.g. membrane bound hydrogenase, Lv-RSN-1, etc., and (4) nucleic acid binding proteins e.g. RNA polymerase holoenzyme, Human PARP-1 bound to a DNA double strand break, etc. Proteins with structural similarity often have same function.

Next, ligands and ligand-binding sites for all five Newt proteins are predicted (Fig 1A''–1E''; S3 Table). These proteins have highest probability to bind with compounds that are involved in redox reactions, and small peptides that might be involved in signaling. Lastly, GO terms based on molecular function, biological process, and cellular component for the corresponding Newt proteins are predicted (S4 Table). With reference to the cellular component, these proteins could be part of cell membrane. In terms of the molecular function, and biological process these proteins may be involved in transport of ions, solute etc., and establishing redox state of the cell. Overall, our results demonstrate that these Newt proteins could be involved in oxidation-reduction reaction, and/or maintaining electric gradient across the cell membrane.

#### Transcriptomics in *Drosophila*

**Fig 2:**
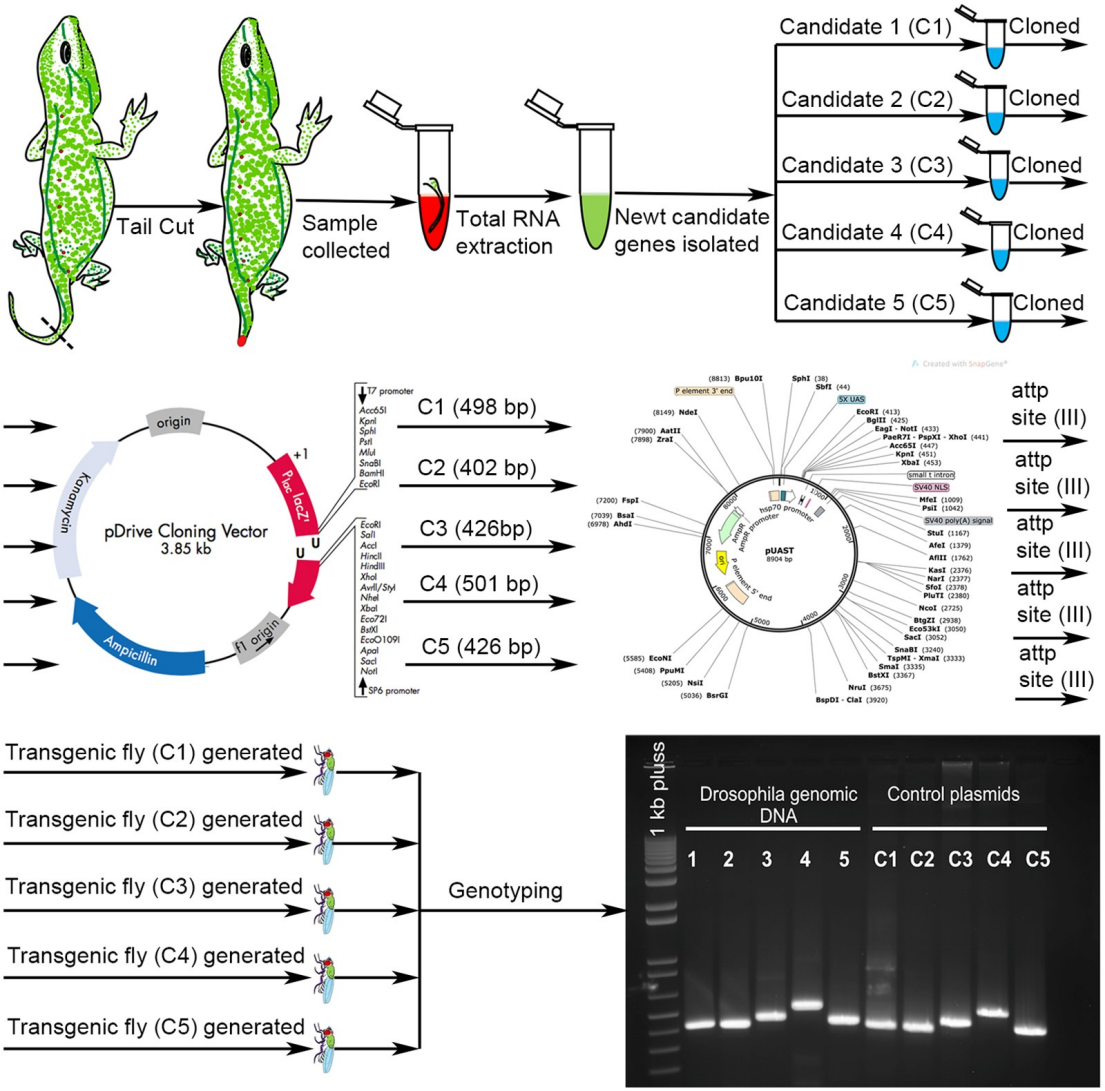
Schematic representation of generation of transgenic Drosophila. Novel Newt candidate genes were used to create 5 transgenic flies. Each fly had a single copy of newt candidate gene (C1, C2, C3, C4 or C5) inserted at chromosome III. After generating transgenic flies, genotyping was performed to check if Newt genes have been properly incorporated in Drosophila genome.

*Notophthalmus viridescens*, Newt, an organism with strong regeneration potential, present challenges with respect to transgenic approaches [11]. *Drosophila*, a genetically tractable model, has been extensively used to express foreign proteins using transgenic approaches [11, 16, 19, 20, 35, 36]. We generated five independent transgenic fly strains for the five Newt genes. We cloned these genes in the UAS- vector and then microinjected in the fly embryo to generate transgenic flies harboring the Newt genes. These transgenic flies were tested to verify the insertion of Newt cDNA in the *Drosophila* genome (Fig 2).

**Fig 3:**
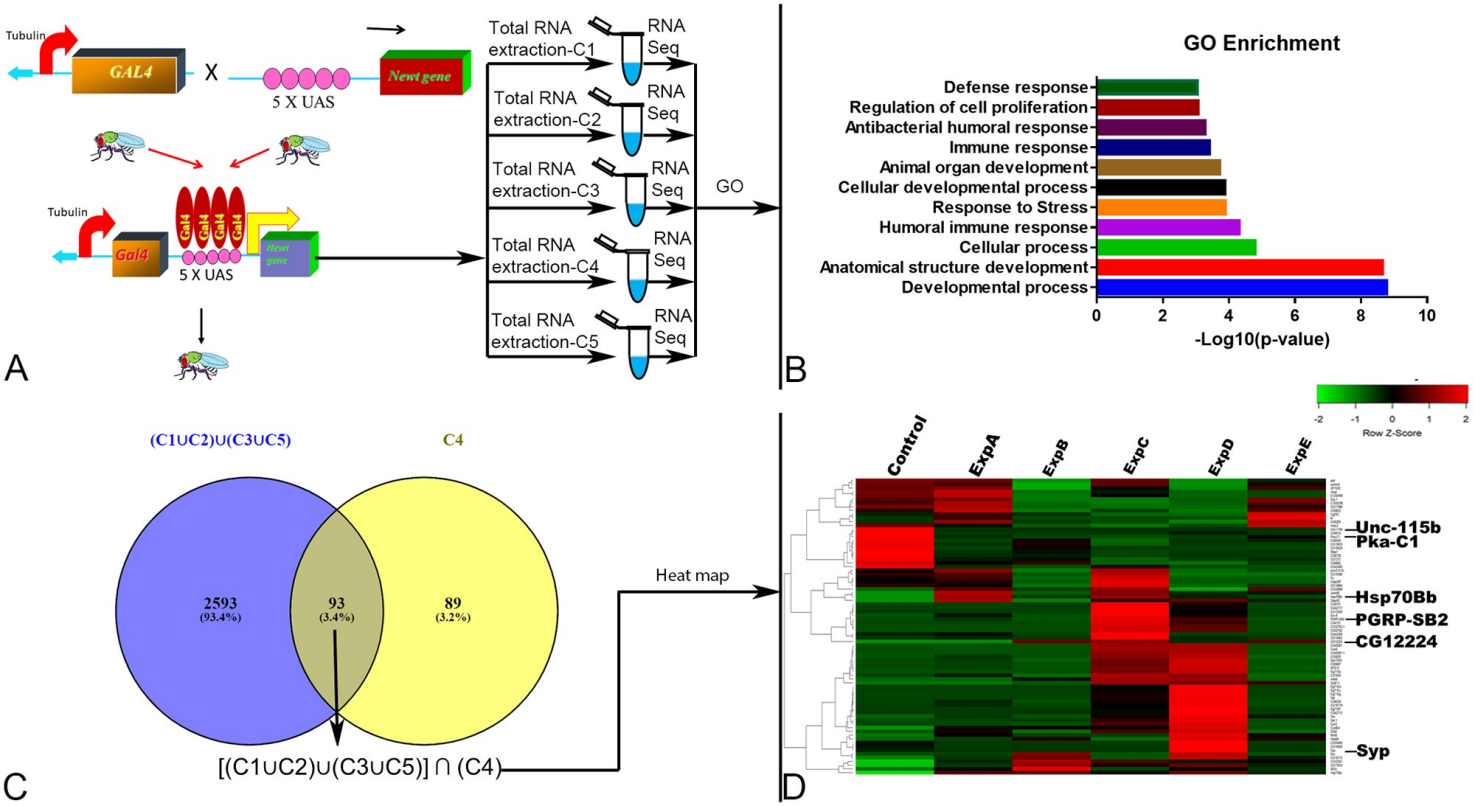
Targeted misexpression of newly identified Newt gene family in Drosophila using Gal4/UAS binary system followed by next generation RNA sequencing. (A) Schematic representation of misexpressing Newt genes ubiquitously in Drosophila using Tubulin driver. It was followed by collection of sample for RNA sequencing at 3^rd^ instar stage [29]. (B) Bar graph showing enriched gene ontology (GO) terms on the scale of–Log_10_ (p-value). (C) Venn diagram showing 93 selected genes (D) Heat map constructed (for 93 common transcripts) from the expression profiles of the 6 sequenced samples. Control: Tubulin Gal4/S-T, Exp A: Candidate 1/Control, ExpB: Candidate 2/Control, ExpC: Candidate 3/Control, ExpD: Candidate 4/Control, ExpE: Candidate 5/Control respectively. Heat map was divided into cluster A: showing genes upregulated by misexpression of C4, and Cluster B: showing genes downregulated by misexpression of C4. The location of the genes used for RT- qPCR analysis is shown on the heat map.

In addition, we wanted to exploit the targeted misexpression approach in *Drosophila* [24] to ectopically express these Newt genes, which encodes the five novel Newt proteins with regeneration potential. The rationale of these experimental approaches was to misexpress these Newt genes in flies and then identify the downstream targets of these five novel Newt genes using next generation RNA sequencing (Fig 3). Since the genetic machinery is highly conserved, the information generated can be extrapolated to other organisms.

#### RNA sequencing supports I-TASSER findings, and show enrichment of genes involved in development, cellular, and immune processes

Sample for RNA sequencing was collected at third instar larval stage during which major developmental events takes place in *Drosophila*. Of the total 36,099 transcripts in *Drosophila*, 34,967 transcripts were detected [37], and 2775 transcripts were significantly regulated. The cohort of genes that were differentially regulated by Newt proteins were grouped according to their biological function. This data suggests that highly enriched genes belong to the category of developmental process like anatomical structure development, cellular development, and organ development; cellular process *e*.*g*. cell cycle and apoptosis; and immune response like humoral immune response, and antibacterial immune response (Fig 3B; S1 Excel File). Upon comparison among the Newt proteins, out of the 2775 transcripts, C1 regulated 2220 transcripts; C2 regulated 1383 transcripts; C3 regulated 1446 transcripts; C4 regulated 182 transcripts; and C5 regulated 2212 transcripts (S2 Excel File). Some of the regulated genes were found to be specific to an individual Newt protein i.e. C1 has 167 (6.30%), C2 has 91 (3.80%), C3 has 146 (4.20%), C4 has 89 (3.20%), and C5 has 153 (5.50%). Very few transcripts [8 transcripts (0.28%)] were common among all 5 Newt proteins (S3 Excel File). Additionally, we selected 93 transcripts that were regulated by C4, and at least by one or more of the other Newt proteins (C1, C2, C3, and C5) [Represented mathematically as: [(C1 ∪ C2) ∪ (C3 ∪C5)] ∩ (C4)]] (Fig 3C; S4 Excel File). Selected 93 transcripts, which are shown as Venn diagram (Fig 3C), are clustered and visualized as a heat map (Fig 3D; S4 Excel File). The visual inspection of the heat map suggests that these novel Newt proteins differentially regulates the *Drosophila* genome.

**Fig 4:**
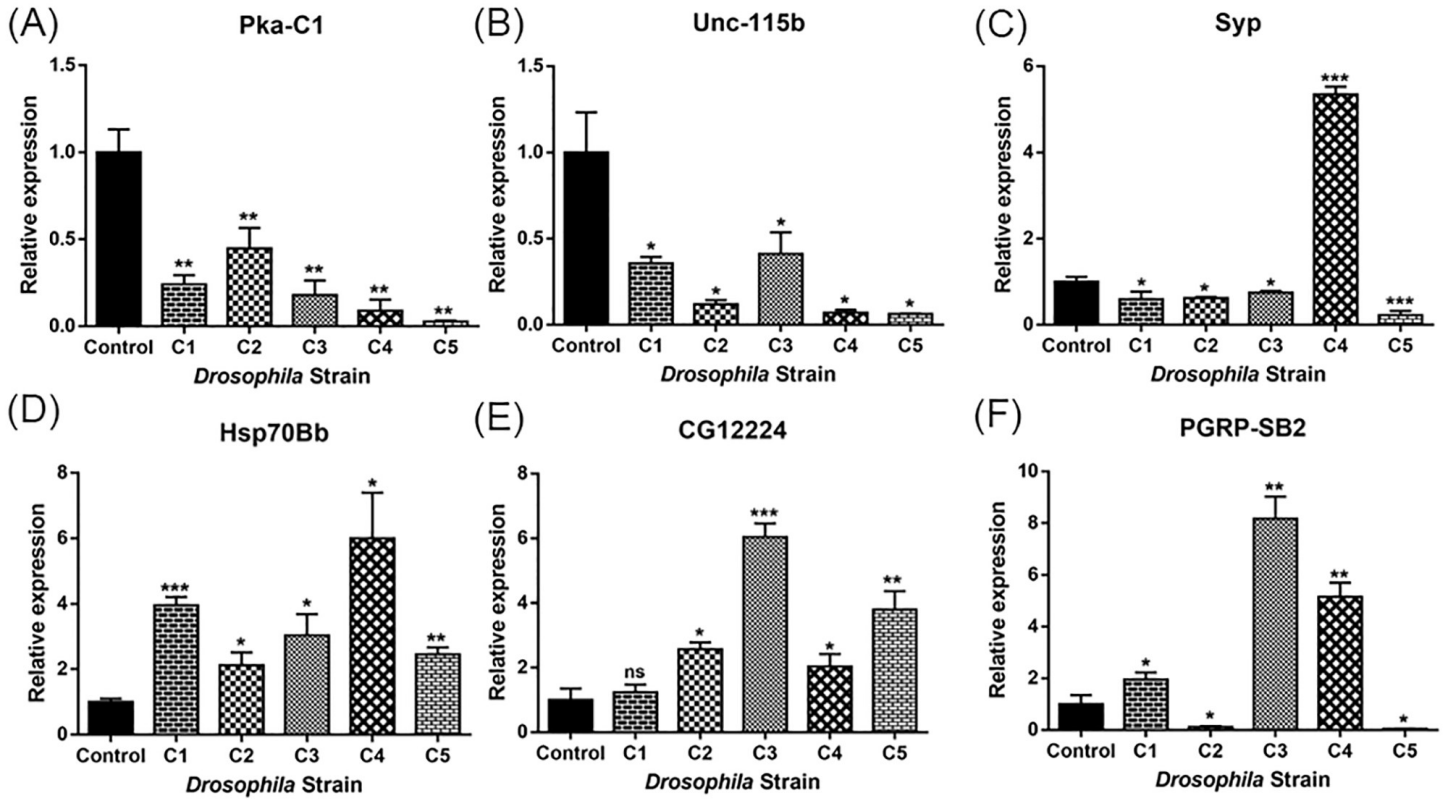
RT-qPCR expression validation of (A) Pka-C1 (B) Unc-115b (C) Syp (D) hsp70Bb (E) CG12224 (F) PGRP-SB2. Expression of the different genes at the RNA level is indicated as relative expression. Bars indicate standard deviation. Statistical test was performed with two-way ANOVA and Student’s t-test. Asterisks above the bars indicate statistical significance (*: p<0.05, **: p<0.005, ***: p< 0.0005) between control: Tublin Gal4/S-T and experimental: C1, C2, C3, C4, and C5 respectively). Total RNA was extracted from 5 larvae for each sample on same day.

Furthermore, enriched gene ontology terms were also calculated for all 2775 transcripts, and individually for transcripts particular to a Newt protein (C1: 167, C2: 91, C3:146, C4: 89, and C5: 153). Each of the data generated almost same results signifying that all these 5 Newt proteins, belonging to the same protein family, have similar functions. As molecular function, genes show enrichment for catalytic activity, binding, transporter activity, and structural molecule activity respectively. In terms of the cellular component- cell, organelles, protein containing complex, membrane, and extracellular complex respectively. In protein class- hydrolase, oxidoreductase, transporter, cytoskeletal protein, nucleic acid binding, transferase, enzyme modulator, and calcium binding protein respectively. Overall, data supported the I-TASSER server finding, and suggested that these Newt proteins may be involved in redox reaction, and transporter activity. RNA sequencing data also reported enrichment of genes belonging to developmental, cellular and immune processes. RNA sequencing data was validated using RT-qPCR for the following set of genes (belonging to selected 93 genes): Pka-C1, hsp70Bb, PGRP-SB2, CG12224, Syp, Unc-115b (Fig 4).

## Discussion

Newts have extraordinary regeneration capability, but it has been studied less than other model organisms in recent decades. This is because of comparatively long reproductive cycle of Newts, their enormous genome size (estimated to reach c × 10^10^ bases), which is about 10-times the size of the human genome [12], and unavailability of genetic tools like generation of transgenic animals in Newts Notophthalmus viridescens [11]. Recent efforts in the field have been directed towards somewhat genetically amenable Newt, the spanish Newt (*Pleurodeles waltl*), which has a shorter life cycle and its genome has been sequenced recently [38]. These Newts are also amenable to transgenesis and CRISPR Cas9 gene editing [38, 39]. Similarly, other highly regenerative animal model that is genetically amenable to manipulations is Zebrafish [40–43]. However, both Spanish Newt as well as Zebrafish have some limitations. The life cycle of Spanish Newt is about 3 months [44], and Zebrafish has about 10–12 weeks [45–47]. Furthermore, both Spanish Newt and Zebrafish model lack a large repository of mutants and transgenic animals are also not readily available [48]. Furthermore, other highly regenerative animals like Hydra, and Planaria also face some challenges with respect to the genetic tools due to lack of powerful tools like GAL4/UAS system to target misexpression of foreign genes [11]. However, *Drosophila*, an insect, has proved to be genetically tractable due to the availability of plethora of genetic tools [15, 16]. Insects exhibit varying range of regeneration potential during development [49–51]. *Drosophila*, which has shown regeneration potential, has a short life cycle of 12 days [15, 52, 53], and a large repository of mutants and transgenic animals are available [17, 54]. In addition, variety of tools to misexpress foreign genes in a spatio-temporal manner are available [55]. Genetic mosaic techniques are widely used to induce genetic changes in a subset of cells or tissues in an individual organism in order to study function of an embryonic lethal gene (sometimes misexpressing foreign genes can be lethal to the organism) [56]. *Drosophila* genome is sequenced [57], and approximately 75% of known vertebrate genes have a recognizable match in the genome of fruit flies. Like Newt and animal models with strong regeneration potential, many evolutionarily conserved pathways like Wg/Wnt, JAK/STAT, Notch, Hedgehog etc. have been reported to promote growth and regeneration in *Drosophila* [58–62]. Therefore, it is expected that the pathways that might get modulated in *Drosophila* by these *Newt genes* can share parallels with their mechanism of action in Newts [11]. Thus, *Drosophila* can serve as a suitable model organism available to address questions pertaining to investigating the function of unique genes from highly regenerative species, which could otherwise be difficult or time consuming to answer by using highly regenerative animal models [16, 35, 53].

Here we provide insight into the function of five newly identified Newt genes that could be involved in redox reaction, and may act as ion transporters. Previously, using microarray analysis, it has been reported that reactive oxygen species (ROS) and mitochondria- related proteins linked to redox system of the cell were highly enriched during early stages of lens regeneration in Newts [21]. Earlier, a significant regulation of these five novel Newt proteins has been shown during early stages of lens regeneration [12]. It suggests that these proteins may play an important role during initial stages of regeneration in Newts. It has been reported that ion transport proteins (bioelectricity), and redox reaction [reactive oxygen species (ROS)] affect downstream biochemical cascades and transcriptional processes influencing biological processes like regeneration, development, and cancer [63–66]. We found that expressing these five Newt genes in *Drosophila* resulted in significant enrichment of genes that are involved in anatomical developmental process, cellular developmental process, and organ developmental process. It also included the members of cellular process (cell cycle, apoptosis etc.) and immune system (humoral immune response, antibacterial humoral response, defense response etc.). It will be interesting to see in future how these novel Newt proteins affect downstream cascade of signaling pathways resulting in differential regulation of cohort of genes.

In Newts, and other animal models like *Drosophila*, apoptotic cells at the site of injury are able to stimulate neighboring surviving cells to undergo additional proliferation [67, 68]. Heat shock protein 70 (Hsp70), which is involved in apoptosis, is an interesting candidate, which is significantly upregulated in all the tested samples. Misexpressing these Newt genes (C1, C2, C3, C4, and C5) showed Hsp70 upregulation of 6.16, 4.69, 6.28, 4.57, and 4.29 fold change respectively (S4 Excel File). Hsp70, and other heat shock proteins like Hsp 90 have been reported as one of the downstream targets getting regulated by difference in redox states of the cell e.g. ROS [64, 69]. It suggests that Newt proteins, which modulate redox state of the cell, may affect heat shock proteins. This integrative interplay may facilitate regeneration in Newts. Previously, it has been reported that Heat shock protein 70 (Hsp70) functions as a chaperone during periods of cellular stress and induces the expression of several inflammatory cytokines that play key role during early liver regeneration in mouse [70]. Hsp70 has also been reported to play role during early stages of *Paramisgurnus dabryanus* fin regeneration [71].

Previously it has been reported in *Drosophila* that cell cycle re-entry of quiescent precursor cells can promote regeneration [72]. Hippo pathway is one of the important pathways that control cell proliferation in *Drosophila* [73–76], and regulate differentiation [77]. YAP and TAZ are the two main downstream effectors of the Hippo pathway, and they function as transcription co-activators to promote cell proliferation and inhibit apoptosis [78]. Phosphorylation of YAP/TAZ by the Lats kinases results in their cytoplasmic retention and ubiquitin-mediated degradation results in inhibition of YAP/TAZ [79]. It is known that cyclic adenosine monophosphate (cAMP), a second messenger downstream from Gα_s_-coupled receptors, acts through protein kinase A (PKA) and Rho GTPases to stimulate Lats kinases and YAP phosphorylation [80]. In our RNA sequencing data we found PKA-C1 significantly downregulated in all five transgenic flies. C3, and C4 showed the maximum fold change downregulation of about -4.95, and -3.82 respectively followed by C2: -3.73, C1: -2.75, and C5: -1.63(S4 Excel File).

The genes belonging to the list of immune system were found to be highly enriched with -log_10_ (p-value)> 4 (Fig 3B). At the wound site, immune cells not only help to clear debris but also secrete numerous signaling molecules that activate appropriate cell proliferation and differentiation programs essential for successful regeneration [81–83]. Finally, the developmental processes are extensively employed during regeneration to rebuild complex, multi-tissue structures in complete polarity [84, 85]. In our studies, we found that genes belonging to the class of developmental process were the most effected (-log_10_ (p-value)> 8).

Previously, regeneration response in *Drosophila* is well studied in wing imaginal discs, leg discs [86], and eye disc [11, 87]. In future, it will be interesting to see if these newly identified Newt genes can promote tissue regeneration in animals with low regeneration potential e.g. *Drosophila*, mammals etc. This study provides important insight into the function of this newly identified Newt protein family, and gives the information about graded expression level of *Drosophila* transcripts after misexpressing Newt proteins.

## Supporting information

S1 Table Residue number of the protein models. Their corresponding (1) predicted Secondary Structure (SS): C—random coil; H—alpha-helix; S—beta-strand, (2) predicted solvent accessibility (SA) at 25% cutoff: E—exposed; B–buried, (3) threading alignment coverage (COV) defined as the number of threading alignments on the residue divided by the number of total threading programs (4) predicted normalized B-factor (BFP), and (5) Residue-Specific Quality (RSQ) defined as the estimated deviation of the residue on the model from the native structure of the protein.
(DOCX)

S2 Table Structural resemblance of novel Newt proteins. Based on high TM-score 10 different proteins with structural resemblance have been reported for each Newt candidate gene. PDB number, RMSD value, and percentage identity between similar proteins is also reported.
(DOCX)

S3 Table Predicted ligand, and ligand binding site of Newt proteins. First two PDB hits, Ligands, C-score, and Ligand binding site residue for all 5 novel Newt proteins have been listed.
(DOCX)

S4 Table Prediction of enriched GO terms. First two consensus prediction of GO terms based on molecular function, biological process, and cellular component for the corresponding Newt candidate proteins have been listed.
(DOCX)

S1 Excel File Enriched GO terms based on Next generation RNA Seq data. The 2775 genes, which were differentially regulated by Newt proteins, were grouped according to their biological function. (Sheet 1) provides list of all developmental process genes, which were differentially regulated, and were further classified according to their function as (1) General developmental process related genes (2) Cellular developmental process (3) Organ developmental process and (4) Anatomical structure development respectively. (Sheet 2) provides list of all cell process related genes that were differentially regulated and were further classified according to their function as (1) Apoptosis related genes, and (2) cell cycle related genes respectively. (Sheet 3) provides list of all immune process related genes, which were differentially regulated, and were further classified according to their function as (1) General immune response related genes (2) Antibacterial immune response related genes and (3) Humoral immune response related genes, respectively. Here ExpA: is Candidate 1 (C1); ExpB: is Candidate 2 (C2); ExpC: is Candidate 3 (C3); ExpD: is Candidate 4 (C4); ExpE: is Candidate 5 (C5); and Control is Tubulin Gal4/SM6-TM6B *Drosophila* stock.
(XLSX)

S2 Excel File Comparative transcriptome analysis. Out of total 34,967 transcripts that were detected (https://doi.org/10.26890/ddlla1a541sgr). (Sheet 1) provides list of all 2775 transcripts that were significantly regulated by misexpressing Newt genes in *Drosophila* under wild type background. Upon comparison among the Newt proteins, out of 2775 transcripts (Sheet 2) C1 regulated 2220 transcripts; (sheet 3) C2 regulated 1383 transcripts; (Sheet 4) C3 regulated 1446 transcripts; (Sheet 5) C4 regulated 182 transcripts; and (Sheet 6) C5 regulated 2212 transcripts respectively. Here ExpA: is Candidate 1 (C1); ExpB: is Candidate 2 (C2); ExpC: is Candidate 3 (C3); ExpD: is Candidate 4 (C4); ExpE: is Candidate 5 (C5); and Control is Tubulin Gal4/ SM6-TM6B *Drosophila* stock.
(XLSX)

S3 Excel File Transcripts exclusive to corresponding newt gene. Some of the regulated transcripts were found to be exclusively modulated by an individual Newt protein (Sheet 1) accession number of transcripts exclusively regulated by C1 (167 (6.30%)), (Sheet 2) accession number of transcripts exclusively regulated by C2 (91 (3.80%)), (Sheet 3) accession number of transcripts exclusively regulated by C3 (146 (4.20%)), (Sheet 4) accession number of transcripts exclusively regulated by C4 (89 (3.20%)), (Sheet 5) accession number of transcripts exclusively regulated by C5 (153 (5.50%)), and (Sheet 6) there were very few transcripts [8 transcripts (0.28%)] that were common among all 5 Newt proteins. Here ExpA: is Candidate 1 (C1); ExpB: is Candidate 2 (C2); ExpC: is Candidate 3 (C3); ExpD: is Candidate 4 (C4); ExpE: is Candidate 5 (C5); and Control is Tubulin Gal4/ SM6-TM6B *Drosophila* stock.
(XLSX)

S4 Excel File Selected transcripts to generate heat map. Selected 93 transcripts that were regulated by C4, and at least by one or more of the other Newt proteins (C1, C2, C3, and C5) that have been selected to generate heat map. Transcripts highlighted in yellow were selected to verify using RT-qPCR. Here ExpA: is Candidate 1 (C1); ExpB: is Candidate 2 (C2); ExpC: is Candidate 3 (C3); ExpD: is Candidate 4 (C4); ExpE: is Candidate 5 (C5); and Control is Tubulin Gal4/ SM6-TM6B *Drosophila* stock.
(XLSX)
