# Supplementary material for: Comparative transcriptomic analysis and structure prediction of novel Newt proteins
Source: PLoS One. 2019 Aug 16;14(8):e0220416. doi: 10.1371/journal.pone.0220416 (PMC6697330; doi:10.1371/journal.pone.0220416)
Supplement: S1 Table — Their corresponding (1) predicted Secondary Structure (SS): C—random coil; H—alpha-helix; S—beta-strand, (2) predicted solvent accessibility (SA) at 25% cutoff: E—exposed; B–buried, (3) threading alignment coverage (COV) defined as the number of threading alignments on the residue divided by the number of total threading programs (4) predicted normalized B-factor (BFP), and (5) Residue-Specific Quality (RSQ) defined as the estimated deviation of the residue on the model from the native structure of the protein. (DOCX) [file pone.0220416.s001.docx]

**S1 Table.** **Residue number of the protein models.** Their corresponding (1) predicted Secondary Structure (SS): C - random coil; H - alpha-helix; S - beta-strand, (2) predicted solvent accessibility (SA) at 25% cutoff: E - exposed; B – buried, (3) threading alignment coverage (COV) defined as the number of threading alignments on the residue divided by the number of total threading programs (4) predicted normalized B-factor (BFP), and (5) Residue-Specific Quality (RSQ) defined as the estimated deviation of the residue on the model from the native structure of the protein.

**Candidate 1**

#RES SS SA COV BFP RSQ_1 RSQ_2 RSQ_3 RSQ_4 RSQ_5

1 C E 0.41 2.07 10.81 13.51 19.27 20.68 14.20

2 C E 0.40 1.28 9.97 11.93 18.14 19.91 12.73

3 S B 0.39 0.54 9.21 10.69 16.99 19.52 10.63

4 S E 0.39 0.18 8.75 10.54 15.58 18.64 10.09

5 S B 0.40 0.04 8.48 10.83 14.63 19.22 10.98

6 S B 0.42 -0.14 8.40 10.99 14.39 20.18 10.55

7 H B 0.42 -0.33 8.04 11.16 12.87 19.70 9.97

8 H B 0.44 -0.34 8.08 11.97 12.07 19.67 10.43

9 C B 0.45 -0.39 9.16 14.19 13.53 20.37 11.05

10 C E 0.47 -0.15 9.85 16.59 15.12 21.41 12.10

11 C E 0.54 0.12 9.81 16.17 15.06 20.86 10.91

12 H B 0.59 -0.08 10.18 17.69 16.27 20.58 12.20

13 H E 0.64 -0.22 9.62 17.73 16.29 20.16 12.14

14 H B 0.63 -0.39 8.51 16.76 15.17 19.06 10.11

15 H E 0.65 -0.24 8.67 17.00 14.94 18.29 10.39

16 H B 0.66 -0.46 9.23 18.77 15.22 17.86 11.35

17 H B 0.66 -0.65 8.78 18.65 14.03 16.56 10.60

18 H B 0.66 -0.65 8.56 17.97 13.19 15.45 9.63

19 H E 0.68 -0.51 9.42 17.73 13.50 15.64 11.29

20 H E 0.71 -0.41 9.49 17.00 13.33 15.55 11.09

21 H B 0.71 -0.52 9.26 16.22 12.96 14.55 10.54

22 H E 0.72 -0.24 9.97 15.60 12.30 14.83 10.66

23 H E 0.72 -0.10 10.48 15.74 11.52 14.08 10.65

24 H E 0.72 -0.23 10.28 15.58 12.27 13.76 10.47

25 H B 0.71 -0.51 10.09 14.25 13.43 13.58 10.97

26 H E 0.74 -0.36 10.29 14.08 12.92 13.83 12.46

27 H E 0.73 -0.36 10.79 14.92 12.30 14.31 12.16

28 H E 0.73 -0.51 10.46 14.46 12.95 14.32 10.17

29 H B 0.74 -0.61 10.12 13.51 12.95 14.20 11.07

30 H E 0.75 -0.47 10.27 13.09 11.71 14.91 12.31

31 H B 0.74 -0.55 9.34 12.77 11.82 15.39 10.16

32 H B 0.73 -0.62 8.43 11.30 11.34 14.81 8.79

33 H E 0.76 -0.56 8.89 11.02 11.28 14.75 10.99

34 H B 0.74 -0.73 8.73 10.82 11.23 13.43 10.78

35 H B 0.71 -0.80 7.94 10.36 10.91 13.48 8.40

36 H B 0.74 -0.80 7.57 9.84 10.31 13.25 8.89

37 H B 0.74 -0.83 8.01 10.03 10.80 12.45 10.68

38 H B 0.73 -0.92 7.73 9.80 11.05 11.94 9.02

39 H B 0.74 -0.92 7.42 10.21 10.00 11.66 8.53

40 H B 0.73 -0.91 8.09 10.74 9.69 11.27 10.67

41 H B 0.73 -0.86 8.17 10.37 10.61 11.36 10.44

42 H B 0.73 -0.83 8.45 10.66 10.69 11.85 9.79

43 H B 0.73 -0.76 8.52 11.90 10.12 11.53 11.25

44 H B 0.72 -0.60 9.34 12.20 10.65 12.19 12.48

45 H B 0.70 -0.42 10.29 12.09 11.70 13.85 11.92

46 C B 0.68 -0.31 10.74 13.40 10.99 13.76 12.85

47 C E 0.65 -0.01 11.13 14.81 11.71 14.36 14.59

48 C E 0.69 0.26 11.83 14.44 12.71 15.26 15.68

49 C E 0.66 0.33 11.98 15.21 13.36 16.28 16.27

50 C E 0.65 0.34 11.29 14.16 13.72 15.47 16.12

51 C E 0.65 0.32 10.90 12.62 12.94 14.48 14.54

52 C E 0.66 0.32 9.90 12.49 12.75 13.94 13.41

53 C E 0.63 0.20 8.76 12.39 13.56 13.94 13.56

54 C E 0.63 -0.05 8.69 11.87 12.27 13.14 12.90

55 C B 0.66 -0.23 9.20 11.81 11.61 13.29 12.15

56 C E 0.67 0.00 8.48 12.17 12.98 13.83 12.53

57 C E 0.66 0.02 7.99 12.50 13.41 13.74 13.72

58 C E 0.65 0.09 9.01 13.19 12.81 13.96 13.29

59 C E 0.64 0.15 9.08 12.94 12.81 13.86 13.70

60 C E 0.64 0.02 8.17 12.20 12.30 13.24 14.32

61 C E 0.66 -0.17 7.80 10.50 10.90 11.59 13.27

62 C B 0.69 -0.13 8.10 10.56 10.30 11.41 13.69

63 C E 0.68 0.23 7.49 12.73 10.97 11.33 13.08

64 C E 0.69 0.24 7.46 12.07 11.07 11.08 12.26

65 C E 0.71 0.12 7.65 11.45 10.44 10.35 12.77

66 C E 0.70 0.17 8.30 12.21 10.47 10.24 13.67

67 C E 0.71 0.09 7.64 11.01 10.55 9.29 14.51

68 H E 0.74 0.01 6.51 9.94 8.98 8.12 13.69

69 H E 0.74 0.04 6.54 10.65 8.12 8.74 13.60

70 H E 0.76 -0.11 6.77 10.45 8.67 8.95 13.19

71 H B 0.77 -0.46 5.75 8.98 7.91 7.71 11.87

72 H B 0.80 -0.47 5.22 9.00 6.73 7.93 10.96

73 H E 0.86 -0.36 5.72 9.27 6.96 8.98 10.59

74 H B 0.84 -0.64 5.57 8.69 7.48 8.73 10.29

75 H B 0.86 -0.64 4.82 7.67 6.36 8.28 8.79

76 H E 0.86 -0.44 5.23 8.17 6.90 9.12 8.96

77 H E 0.88 -0.21 5.78 8.98 8.07 9.52 8.84

78 H B 0.86 -0.35 5.50 8.41 7.81 9.32 8.48

79 H B 0.89 -0.24 5.05 7.98 7.47 9.20 7.91

80 H E 0.89 0.20 5.83 8.72 8.73 10.09 8.57

81 C E 0.83 0.26 6.36 9.67 9.58 10.43 8.93

82 C E 0.81 0.08 6.11 9.85 9.70 11.18 9.79

83 C B 0.81 -0.05 6.16 10.34 9.54 10.43 9.92

84 H E 0.87 0.05 5.96 9.79 8.29 10.09 8.85

85 H E 0.86 -0.08 5.97 9.17 7.28 9.56 7.85

86 H B 0.87 -0.40 5.20 8.17 6.81 8.51 7.49

87 H B 0.89 -0.46 5.25 8.40 7.25 8.72 7.95

88 H E 0.89 -0.25 6.05 8.81 7.13 9.05 7.67

89 H E 0.91 -0.35 5.78 7.91 6.55 8.23 6.77

90 H B 0.90 -0.59 4.79 7.33 6.36 7.35 6.32

91 H B 0.92 -0.54 5.44 8.08 6.93 8.27 6.68

92 H E 0.93 -0.46 6.24 8.44 6.88 8.59 6.73

93 H B 0.94 -0.65 5.44 7.44 6.48 7.61 5.69

94 H B 0.93 -0.72 4.90 7.11 6.81 7.67 6.10

95 H B 0.91 -0.65 6.03 8.11 7.64 8.92 6.83

96 H B 0.91 -0.53 6.41 8.26 7.49 8.99 6.84

97 H B 0.94 -0.41 5.52 7.78 7.59 8.42 6.15

98 H E 0.93 -0.05 5.99 8.12 8.41 9.35 7.23

99 H E 0.91 0.19 7.20 8.73 8.64 10.46 7.90

100 C E 0.89 0.37 7.47 9.06 8.87 10.61 8.42

101 C E 0.91 0.52 7.63 9.96 9.44 11.33 8.95

102 H E 0.90 0.48 7.56 10.65 10.58 11.92 9.59

103 H E 0.94 0.34 7.12 9.16 9.86 10.96 10.56

104 H E 0.91 0.23 6.75 9.19 10.35 10.73 11.51

105 H E 0.93 -0.00 6.25 8.61 10.53 9.96 10.43

106 H E 0.95 -0.11 6.64 7.93 9.80 9.61 10.07

107 H E 0.95 -0.15 7.73 7.87 9.05 9.01 12.21

108 H E 0.91 -0.40 7.29 7.90 7.80 8.93 11.58

109 H B 0.94 -0.60 5.78 7.33 7.75 7.98 8.73

110 H E 0.93 -0.54 6.64 7.19 8.04 7.41 10.43

111 H E 0.94 -0.44 7.62 7.16 7.25 7.77 12.14

112 H B 0.96 -0.65 6.25 6.91 6.41 7.26 9.78

113 H B 0.96 -0.62 5.43 6.52 6.65 6.20 8.78

114 H E 0.96 -0.40 7.30 6.63 6.62 6.75 11.72

115 H E 0.96 -0.39 7.38 6.51 5.98 7.08 11.23

116 H B 0.96 -0.58 5.46 6.46 5.65 6.19 8.32

117 H E 0.96 -0.37 6.59 6.44 6.05 6.25 10.63

118 H E 0.94 -0.35 8.02 6.88 6.21 7.33 12.43

119 H B 0.94 -0.49 6.85 6.99 5.79 7.36 10.24

120 H B 0.92 -0.49 5.76 6.72 5.96 6.88 9.10

121 H E 0.92 -0.21 7.87 7.08 6.59 7.61 12.65

122 H E 0.89 -0.11 8.17 7.50 6.80 8.49 13.16

123 C B 0.84 -0.07 6.63 7.68 7.15 8.40 10.58

124 C E 0.81 -0.10 7.49 7.93 7.50 8.77 11.01

125 C B 0.76 -0.16 6.72 7.00 7.06 7.97 9.85

126 C E 0.83 -0.12 7.69 7.28 6.37 7.22 11.33

127 H E 0.82 0.09 7.83 7.23 6.58 6.34 11.22

128 H E 0.83 0.16 8.82 7.34 6.54 6.92 12.64

129 H E 0.81 -0.07 7.53 7.28 7.12 7.65 10.50

130 H B 0.82 -0.31 5.99 8.08 7.35 7.12 7.30

131 H E 0.82 -0.10 6.82 7.90 7.13 7.32 9.26

132 H E 0.83 -0.08 7.58 7.09 7.04 8.41 10.05

133 H B 0.81 -0.34 6.71 7.77 8.23 9.36 9.08

134 H E 0.86 -0.16 5.81 8.35 9.15 9.22 9.13

135 H E 0.87 -0.13 6.58 8.82 8.30 10.16 10.94

136 H E 0.88 -0.27 6.39 7.32 7.68 9.16 10.40

137 H B 0.86 -0.34 5.82 7.27 9.67 8.89 8.51

138 H E 0.86 -0.12 6.53 8.56 10.14 9.71 10.07

139 H E 0.82 -0.09 6.80 8.30 9.37 10.42 11.96

140 H B 0.81 -0.23 7.32 8.97 11.40 11.49 11.62

141 H E 0.81 -0.05 7.13 8.95 12.75 11.12 11.52

142 H E 0.80 0.19 7.43 8.99 12.71 10.80 11.37

143 H E 0.76 0.13 7.75 8.79 12.05 10.13 10.77

144 C B 0.75 -0.08 7.53 10.62 12.56 11.20 11.70

145 C E 0.72 0.17 7.02 10.50 11.96 11.34 10.20

146 C E 0.69 0.35 6.41 8.16 11.34 9.89 8.54

147 C E 0.66 0.05 6.45 8.78 11.05 8.96 9.16

148 C E 0.65 0.02 5.48 9.21 10.73 9.57 8.45

149 C E 0.65 0.20 5.07 8.67 10.46 11.08 8.62

150 C E 0.60 0.17 5.80 9.42 9.11 9.96 7.75

151 C E 0.59 0.03 5.40 8.74 8.53 9.07 7.91

152 C E 0.57 0.03 4.52 8.38 9.15 9.52 8.02

153 C E 0.56 0.18 5.22 8.72 10.46 10.90 8.53

154 C E 0.53 0.10 5.71 8.92 12.02 12.00 8.61

155 C E 0.46 0.11 5.99 9.15 13.68 12.98 10.23

156 C E 0.43 0.09 6.05 9.74 14.79 14.44 10.92

157 C E 0.40 -0.00 6.55 10.18 15.09 16.70 11.75

158 S E 0.41 -0.24 6.72 10.59 14.90 16.78 11.66

159 S E 0.39 0.01 6.91 11.72 15.80 18.30 12.29

160 S E 0.38 0.09 7.32 13.19 16.50 20.15 12.56

161 C B 0.37 0.14 7.21 14.46 17.08 20.32 12.27

162 C E 0.36 0.40 7.76 16.13 17.56 21.09 13.41

163 C E 0.37 0.80 7.95 17.32 18.12 21.66 13.95

164 C B 0.39 1.14 8.71 18.21 19.77 21.80 14.71

165 C E 0.39 2.02 9.59 19.62 20.91 23.24 15.95

**Candidate 2**

#RES SS SA COV BFP RSQ_1 RSQ_2 RSQ_3 RSQ_4 RSQ_5

1 C E 0.51 1.48 14.45 23.89 21.90 22.74 21.30

2 H E 0.53 0.74 13.47 22.62 20.90 22.01 20.34

3 H B 0.55 0.05 12.92 20.91 19.84 20.51 18.84

4 H B 0.56 -0.46 12.45 19.86 18.63 19.41 17.86

5 H B 0.58 -0.59 11.16 18.74 17.17 18.83 17.01

6 H B 0.58 -0.75 10.41 17.61 16.42 18.13 16.10

7 H B 0.58 -0.87 10.00 16.03 15.70 16.82 15.20

8 H B 0.57 -0.89 9.35 15.18 14.88 16.52 14.35

9 H B 0.59 -0.81 8.55 14.23 13.95 16.12 13.57

10 H B 0.59 -0.73 8.17 12.72 13.53 15.12 12.93

11 H B 0.60 -0.71 7.98 12.32 13.69 14.83 12.58

12 H B 0.61 -0.70 8.00 12.10 13.07 14.73 11.50

13 H B 0.61 -0.59 7.82 12.00 13.01 14.21 11.54

14 H B 0.60 -0.47 7.80 11.77 13.26 14.44 12.21

15 H B 0.59 -0.26 8.21 12.29 13.38 14.91 11.83

16 C B 0.56 -0.22 8.05 11.89 13.23 14.50 11.23

17 C E 0.60 0.08 7.66 11.70 14.32 14.58 12.43

18 C E 0.58 0.29 8.41 12.35 14.88 15.23 12.98

19 C E 0.56 0.31 8.46 12.54 13.90 14.38 12.14

20 C E 0.59 0.32 8.54 12.23 14.30 14.45 12.90

21 C E 0.61 0.42 9.07 12.53 15.07 14.63 13.75

22 C E 0.62 0.37 8.75 12.25 14.49 14.12 12.93

23 C B 0.61 0.10 8.13 11.69 13.02 14.00 12.37

24 H E 0.56 -0.18 7.77 11.89 11.84 14.18 12.71

25 H B 0.57 -0.27 7.25 11.51 12.72 13.68 13.04

26 H E 0.59 -0.12 7.17 10.80 12.61 12.31 12.79

27 H B 0.56 -0.08 7.14 11.49 11.50 12.65 12.80

28 H E 0.56 -0.10 7.44 12.86 12.06 13.48 13.54

29 H E 0.59 -0.01 7.58 12.59 12.38 13.53 14.24

30 C E 0.58 0.02 6.86 11.88 11.54 13.14 13.20

31 C E 0.59 0.19 6.93 12.48 11.55 14.46 13.53

32 C E 0.59 0.21 6.96 11.78 11.94 14.57 12.71

33 C E 0.56 -0.08 7.34 10.30 12.36 13.47 11.86

34 C E 0.58 -0.01 6.98 10.33 12.29 12.88 10.87

35 C E 0.59 0.03 6.06 9.60 10.93 11.19 10.08

36 C B 0.65 -0.12 5.47 8.08 9.46 9.63 9.27

37 H E 0.73 0.04 5.19 7.74 9.97 9.62 9.17

38 H E 0.72 -0.22 5.28 7.79 9.80 9.00 9.48

39 H B 0.75 -0.52 4.94 7.03 8.36 8.08 8.03

40 H B 0.80 -0.48 4.57 7.15 8.08 8.45 7.98

41 H E 0.84 -0.39 4.79 6.91 8.87 8.67 8.88

42 H B 0.84 -0.68 4.78 6.38 8.62 7.90 8.41

43 H B 0.85 -0.71 4.16 6.18 7.68 7.37 7.66

44 H E 0.86 -0.46 4.45 6.86 8.37 8.18 8.94

45 H E 0.86 -0.30 4.95 7.39 9.38 8.30 10.02

46 H B 0.85 -0.38 4.88 7.15 8.75 7.89 9.55

47 H B 0.87 -0.27 4.24 7.13 8.20 7.69 9.47

48 H E 0.88 0.08 4.83 7.91 9.41 8.50 10.85

49 H E 0.88 0.20 5.36 8.33 10.30 8.89 11.68

50 H E 0.88 -0.02 5.47 8.19 9.99 8.61 11.84

51 H B 0.89 -0.26 5.66 8.47 8.96 8.94 10.31

52 H E 0.93 -0.12 5.37 9.01 7.92 8.83 10.44

53 H E 0.94 -0.22 5.11 7.36 7.29 7.62 9.80

54 H B 0.94 -0.56 4.57 6.66 6.87 6.86 8.60

55 H B 0.95 -0.68 4.04 7.05 6.78 6.73 8.05

56 H E 0.96 -0.44 4.37 7.32 6.56 6.85 8.37

57 H E 0.96 -0.46 4.38 6.63 6.13 6.36 7.98

58 H B 0.95 -0.67 3.64 5.98 5.70 5.71 6.76

59 H B 0.96 -0.67 3.55 6.93 5.69 6.27 6.90

60 H E 0.95 -0.57 4.11 7.09 5.84 6.34 7.38

61 H B 0.94 -0.75 3.84 6.15 5.37 5.50 6.70

62 H B 0.94 -0.79 3.44 6.53 5.29 5.50 6.04

63 H B 0.94 -0.70 3.86 7.73 5.80 6.34 6.84

64 H E 0.95 -0.56 4.03 7.59 5.84 6.33 7.33

65 H B 0.95 -0.43 3.88 7.33 5.78 6.15 7.03

66 H E 0.95 -0.10 3.98 8.24 6.69 6.78 7.35

67 H E 0.96 0.10 4.48 9.23 7.37 7.26 8.18

68 C E 0.95 0.27 4.61 9.59 7.45 7.56 9.18

69 C E 0.94 0.39 5.30 10.36 8.46 8.43 9.95

70 H E 0.87 0.45 6.00 11.22 9.59 9.39 10.82

71 H E 0.90 0.39 5.28 10.45 8.40 9.15 10.38

72 H E 0.93 0.27 5.07 9.65 8.62 8.74 10.22

73 H E 0.92 0.10 5.29 9.53 8.48 7.80 10.08

74 H E 0.94 0.02 5.07 8.91 7.96 7.67 10.04

75 H E 0.95 -0.03 5.40 8.50 7.29 7.40 9.73

76 H E 0.95 -0.30 4.98 8.12 6.67 7.15 9.20

77 H B 0.97 -0.56 4.32 7.43 6.15 6.29 8.05

78 H E 0.96 -0.45 4.76 7.26 6.58 6.54 8.26

79 H E 0.96 -0.32 4.99 7.00 6.51 6.48 8.45

80 H B 0.96 -0.53 4.28 6.40 5.84 5.56 7.39

81 H B 0.96 -0.56 4.27 6.03 5.56 5.44 7.03

82 H E 0.96 -0.37 5.12 6.25 6.12 5.91 7.84

83 H E 0.97 -0.34 4.87 5.84 6.00 5.66 7.70

84 H B 0.99 -0.50 4.23 5.16 5.59 5.09 6.64

85 H E 0.97 -0.36 5.09 5.64 6.12 5.68 7.33

86 H E 0.96 -0.35 5.53 6.09 6.62 6.24 8.27

87 H B 0.96 -0.50 4.86 5.63 6.43 6.05 7.92

88 H B 0.94 -0.55 4.73 5.64 6.51 6.09 7.78

89 H E 0.94 -0.31 5.66 6.66 7.00 6.83 8.74

90 H E 0.92 -0.10 5.89 7.10 7.62 7.22 9.62

91 C B 0.89 -0.10 5.43 6.91 7.90 7.45 9.72

92 C E 0.89 -0.09 5.72 7.27 8.18 7.79 9.80

93 C B 0.82 -0.23 5.12 6.71 7.76 7.30 8.30

94 C E 0.86 -0.06 5.47 6.85 7.21 7.09 8.07

95 H E 0.88 -0.03 5.28 6.25 6.67 6.87 7.41

96 H E 0.88 -0.09 5.86 7.22 6.82 7.68 7.66

97 H B 0.86 -0.32 5.43 7.07 7.03 8.35 7.42

98 H B 0.88 -0.39 5.30 6.75 6.69 8.20 7.05

99 H E 0.88 -0.11 5.92 8.14 6.97 9.79 7.47

100 H E 0.86 -0.04 6.50 8.83 7.22 10.44 7.75

101 H B 0.86 -0.23 6.69 8.90 8.62 10.58 9.36

102 H B 0.89 -0.15 6.45 8.94 8.41 10.47 9.14

103 H E 0.90 0.07 6.63 9.06 8.37 11.25 8.99

104 H E 0.91 -0.07 6.26 9.53 7.24 10.86 8.24

105 H B 0.90 -0.31 6.59 9.90 8.30 11.12 8.63

106 H E 0.91 -0.21 5.71 9.10 7.50 10.86 8.47

107 H E 0.91 -0.21 5.11 8.49 6.97 8.82 7.86

108 H B 0.87 -0.48 4.87 8.42 6.83 7.59 7.39

109 H B 0.88 -0.46 4.31 7.34 5.64 7.75 6.96

110 H E 0.89 -0.18 4.07 7.78 5.94 7.35 6.90

111 H E 0.89 -0.16 4.19 7.86 5.99 6.90 6.43

112 H B 0.87 -0.36 3.84 6.72 5.47 6.08 5.90

113 H E 0.89 -0.25 3.44 6.59 5.48 5.79 6.02

114 H E 0.89 -0.14 3.64 7.46 5.91 6.93 6.07

115 H E 0.88 -0.27 3.78 7.08 6.08 7.25 6.23

116 H B 0.88 -0.52 3.21 6.21 5.72 6.45 5.87

117 H B 0.87 -0.42 3.19 7.14 6.12 6.84 6.04

118 H E 0.87 -0.23 3.77 7.75 6.68 7.83 6.76

119 H B 0.87 -0.45 3.46 7.03 6.73 7.55 6.95

120 H B 0.88 -0.43 3.15 7.06 6.71 7.10 7.28

121 H E 0.86 -0.03 3.66 7.99 7.53 8.08 7.60

122 H E 0.86 0.03 3.98 8.10 7.97 8.77 8.49

123 C B 0.84 -0.10 3.84 7.56 8.08 8.48 8.95

124 C E 0.74 -0.02 4.33 8.54 8.53 9.03 9.47

125 C E 0.66 0.19 4.18 8.27 8.03 8.44 9.00

126 C E 0.60 0.23 4.91 9.14 8.60 8.97 9.52

127 C E 0.55 0.35 5.47 9.46 8.55 9.39 9.19

128 C E 0.54 0.42 5.92 9.81 9.70 10.62 9.61

129 C E 0.49 0.38 6.70 10.89 10.18 11.16 10.60

130 C E 0.43 0.66 7.05 11.87 10.28 10.64 10.98

131 C E 0.40 1.01 7.36 12.64 11.00 11.74 11.42

132 C E 0.31 1.30 8.23 13.50 12.10 13.15 12.28

133 C E 0.30 2.15 9.33 15.18 13.23 14.08 13.91

**Candidate 3**

#RES SS SA COV BFP RSQ_1 RSQ_2 RSQ_3 RSQ_4 RSQ_5

1 C E 0.55 2.29 13.70 18.96 18.29 19.43 20.63

2 C E 0.59 1.39 12.99 17.38 17.55 18.00 19.86

3 S B 0.60 0.55 11.87 15.73 15.95 16.91 18.78

4 S E 0.62 0.25 10.63 15.34 15.24 15.36 16.96

5 H B 0.61 0.24 10.24 14.75 15.71 14.13 15.75

6 H B 0.63 -0.22 9.70 12.94 15.10 14.07 15.94

7 H B 0.63 -0.34 8.57 12.16 13.61 13.42 14.69

8 H B 0.63 -0.35 8.02 11.86 13.57 12.14 12.82

9 H B 0.63 -0.37 8.07 10.61 14.42 12.12 12.94

10 H B 0.62 -0.31 7.79 10.48 13.71 12.86 13.54

11 H B 0.63 -0.20 7.85 10.40 12.83 13.20 12.61

12 H B 0.64 -0.03 8.10 9.76 13.44 13.18 12.08

13 H E 0.64 0.07 8.08 10.18 13.85 13.85 13.32

14 C B 0.60 -0.13 7.67 10.81 12.89 15.02 14.10

15 C E 0.61 0.12 7.92 12.07 12.77 15.62 14.51

16 C B 0.61 0.11 8.92 12.20 13.48 15.63 15.35

17 C E 0.64 0.25 8.95 13.53 12.99 16.61 15.38

18 C E 0.64 0.16 9.02 14.03 11.84 16.42 15.62

19 C E 0.64 0.04 9.41 13.59 12.46 14.78 15.15

20 C E 0.66 0.11 9.54 12.00 12.97 14.01 15.11

21 C E 0.69 0.19 9.98 11.41 12.90 14.24 15.00

22 C E 0.69 0.11 10.39 11.78 12.37 13.79 15.54

23 C B 0.68 -0.20 9.94 11.82 11.99 12.43 14.93

24 H B 0.69 -0.38 9.69 11.77 12.53 11.13 14.25

25 H B 0.69 -0.40 10.34 11.16 13.34 12.27 13.59

26 H B 0.67 -0.49 10.88 12.55 12.60 12.23 12.71

27 H B 0.68 -0.45 10.54 12.59 12.37 11.17 11.80

28 C B 0.66 -0.35 10.52 12.25 13.24 11.64 11.74

29 C B 0.66 -0.09 10.75 13.12 13.71 12.76 11.79

30 C E 0.67 0.23 11.08 13.55 14.67 13.85 11.34

31 C E 0.65 0.31 11.39 14.41 15.22 15.70 11.50

32 C E 0.64 0.17 11.57 16.71 16.08 15.90 11.75

33 C E 0.64 0.05 11.15 15.92 15.29 15.53 11.70

34 C B 0.66 0.00 10.16 13.06 14.25 15.83 10.82

35 H E 0.66 -0.01 9.31 13.30 14.82 14.67 10.47

36 H B 0.69 -0.22 8.53 13.28 12.97 12.78 10.20

37 H E 0.69 -0.30 8.49 11.41 11.48 13.18 10.93

38 H E 0.69 -0.22 8.13 9.88 11.65 15.78 10.86

39 C E 0.69 -0.25 7.43 10.96 12.29 15.34 10.00

40 C B 0.69 -0.48 7.21 10.78 11.00 13.83 11.31

41 H E 0.71 -0.44 7.02 10.21 11.12 13.25 12.17

42 H B 0.73 -0.41 5.98 8.02 10.91 11.64 10.31

43 H B 0.75 -0.54 5.35 8.53 9.90 11.40 9.32

44 H B 0.75 -0.70 5.53 9.20 10.85 11.68 10.53

45 H B 0.76 -0.68 5.97 8.56 10.55 11.62 10.22

46 H B 0.84 -0.67 6.53 7.09 9.53 12.06 8.75

47 H E 0.86 -0.61 6.56 7.12 10.19 12.52 8.27

48 H B 0.86 -0.73 5.79 6.33 9.06 10.36 8.09

49 H B 0.86 -0.70 6.67 8.22 7.52 10.18 7.17

50 H E 0.86 -0.48 7.80 8.42 8.14 11.18 7.65

51 H E 0.84 -0.44 7.08 6.65 8.90 9.85 8.55

52 H B 0.84 -0.65 6.59 7.30 7.45 9.27 8.87

53 H E 0.82 -0.50 7.88 8.84 7.64 9.20 9.27

54 C B 0.82 -0.48 8.14 7.94 7.71 9.28 10.03

55 H E 0.85 -0.34 7.70 7.57 7.79 9.02 11.26

56 H E 0.89 -0.51 7.32 7.54 7.91 8.50 10.95

57 H B 0.90 -0.57 6.36 8.79 8.59 8.58 11.35

58 H B 0.91 -0.44 6.24 8.13 8.09 8.36 11.69

59 H E 0.91 -0.36 6.03 7.68 7.89 7.22 10.48

60 H B 0.92 -0.63 5.28 6.81 6.73 6.71 9.29

61 H B 0.94 -0.63 5.42 6.87 6.44 6.57 7.90

62 H E 0.94 -0.38 5.66 7.13 7.04 6.51 8.39

63 H E 0.94 -0.42 4.83 6.62 6.33 6.09 8.70

64 H B 0.94 -0.57 4.53 6.35 5.46 5.74 7.23

65 H E 0.94 -0.46 5.18 6.89 6.61 6.03 6.97

66 H E 0.93 -0.46 5.00 7.14 6.85 6.07 8.17

67 H B 0.91 -0.73 4.56 6.73 6.11 5.48 8.13

68 H B 0.91 -0.83 4.71 6.98 6.48 5.52 7.08

69 H B 0.88 -0.70 5.28 7.64 7.62 6.19 7.90

70 H B 0.83 -0.68 5.21 7.87 7.72 6.13 9.35

71 H B 0.89 -0.59 4.91 7.77 7.48 5.52 8.85

72 H B 0.89 -0.30 5.36 8.58 8.54 6.14 9.04

73 C B 0.89 -0.15 5.79 8.90 9.29 6.49 10.24

74 C E 0.86 0.21 5.80 9.10 9.18 6.48 10.67

75 C E 0.87 0.36 6.52 10.47 11.16 7.46 11.20

76 C E 0.86 0.26 6.73 11.17 11.93 7.88 11.41

77 H B 0.85 0.18 6.45 10.62 10.48 7.74 10.25

78 H E 0.81 0.36 6.75 9.36 9.87 8.85 10.86

79 H E 0.86 0.26 6.71 8.09 8.98 8.59 10.55

80 H E 0.87 0.07 6.76 8.55 8.05 8.27 9.63

81 H E 0.88 0.02 6.51 8.50 7.60 7.84 9.62

82 H E 0.87 -0.22 5.73 7.21 7.38 7.41 9.63

83 H B 0.90 -0.54 5.00 7.00 6.59 7.17 8.54

84 H B 0.94 -0.50 5.25 7.12 6.42 7.28 8.76

85 H E 0.94 -0.42 5.08 6.64 6.61 7.10 8.98

86 H B 0.94 -0.57 4.30 6.01 6.22 6.47 8.47

87 H B 0.94 -0.63 4.08 5.93 6.02 6.45 8.35

88 H E 0.94 -0.34 4.68 6.18 6.64 6.82 9.08

89 H E 0.92 -0.39 4.32 6.04 6.16 6.60 9.06

90 H B 0.92 -0.62 3.88 5.30 5.68 5.89 7.91

91 H E 0.92 -0.44 4.34 5.68 7.03 6.61 9.02

92 H E 0.95 -0.30 4.66 5.95 7.38 7.01 10.38

93 H B 0.95 -0.57 4.58 5.78 6.55 6.43 9.57

94 H B 0.94 -0.57 4.74 5.91 6.94 6.39 8.81

95 H E 0.94 -0.23 5.20 6.43 8.48 7.32 10.91

96 H E 0.93 -0.16 5.72 7.33 8.64 7.56 11.73

97 C B 0.87 -0.21 5.88 7.64 8.41 7.52 10.32

98 C E 0.85 -0.19 6.09 8.02 9.70 7.88 11.39

99 H B 0.85 -0.36 5.46 7.52 9.47 7.55 11.09

100 H B 0.91 -0.45 5.29 7.32 9.24 6.95 11.12

101 H B 0.92 -0.33 5.18 7.35 8.17 7.43 9.93

102 H E 0.92 -0.24 5.02 8.19 9.64 8.37 10.88

103 H B 0.94 -0.48 5.73 7.51 10.24 7.98 11.15

104 H B 0.94 -0.48 5.91 8.68 9.36 7.98 9.58

105 H E 0.94 -0.36 5.81 8.57 10.02 9.00 9.37

106 H B 0.89 -0.47 6.07 8.25 11.26 9.78 11.75

107 H B 0.93 -0.60 6.70 8.75 11.71 9.55 12.68

108 H E 0.93 -0.41 7.27 10.04 12.57 9.99 12.09

109 H E 0.92 -0.23 7.27 10.44 12.07 11.09 11.21

110 H E 0.92 -0.40 6.97 9.92 10.59 11.57 10.72

111 H B 0.93 -0.48 7.17 10.72 10.24 10.60 11.86

112 H E 0.94 -0.35 7.25 11.46 11.18 8.78 11.19

113 H E 0.92 -0.36 6.12 10.45 10.03 8.30 9.22

114 H B 0.91 -0.58 5.31 8.42 9.53 8.10 8.87

115 H B 0.91 -0.53 4.60 7.48 8.00 6.71 7.34

116 H E 0.91 -0.26 4.74 9.00 8.53 6.53 7.18

117 H E 0.89 -0.19 5.12 9.87 9.55 7.31 7.87

118 C B 0.89 -0.36 4.84 9.74 8.54 6.86 7.06

119 C E 0.87 -0.23 4.60 9.36 7.74 6.04 5.93

120 C E 0.87 -0.21 5.19 9.88 9.21 6.59 6.73

121 C E 0.85 -0.32 5.07 8.99 9.68 6.95 7.06

122 H B 0.88 -0.50 4.42 7.74 8.08 6.14 5.92

123 H B 0.87 -0.44 5.15 8.90 8.13 6.15 5.75

124 H E 0.88 -0.27 5.58 8.68 9.83 7.06 6.68

125 H B 0.86 -0.41 5.14 7.23 9.43 6.88 6.39

126 H B 0.85 -0.39 5.37 8.16 8.17 6.50 5.93

127 H E 0.83 -0.01 6.23 9.42 9.77 7.50 6.78

128 H E 0.75 0.06 6.45 8.82 10.93 7.73 7.16

129 C B 0.78 -0.14 6.44 8.73 9.49 7.50 6.91

130 C E 0.68 -0.02 7.26 10.84 9.66 7.98 7.65

131 C B 0.66 -0.05 7.82 10.87 8.71 8.66 9.02

132 H B 0.71 -0.19 7.38 10.62 8.80 8.36 9.41

133 H E 0.69 -0.15 7.38 10.98 9.69 8.14 8.56

134 H B 0.68 -0.21 7.46 12.16 10.33 7.96 9.74

135 H B 0.66 -0.29 7.12 11.38 8.76 7.89 9.64

136 H B 0.67 -0.10 7.12 12.16 9.18 9.64 9.60

137 H B 0.64 0.06 7.56 14.70 11.21 10.98 11.25

138 H B 0.61 0.23 7.77 15.33 11.53 11.86 11.98

139 C E 0.57 0.75 7.93 14.94 10.88 12.88 12.28

140 C E 0.54 1.26 8.46 17.06 12.79 14.51 13.97

141 C E 0.54 2.23 9.01 19.12 14.51 16.08 15.35

**Candidate 4**

#RES SS SA COV BFP RSQ_1 RSQ_2 RSQ_3 RSQ_4 RSQ_5

1 C E 0.61 1.87 14.57 15.17 23.89 16.27 20.96

2 H E 0.66 0.89 13.63 14.81 22.85 14.11 18.74

3 H B 0.67 0.28 13.26 14.26 21.45 13.28 17.81

4 H B 0.69 -0.16 11.87 13.91 20.26 14.80 17.35

5 H B 0.58 -0.31 10.69 14.36 19.97 14.42 16.16

6 H B 0.70 -0.50 10.77 13.17 18.90 11.87 14.26

7 H B 0.70 -0.63 10.64 12.81 17.58 12.68 14.20

8 H B 0.71 -0.73 8.97 13.35 16.97 13.83 13.76

9 H B 0.71 -0.69 8.44 13.56 17.61 12.03 12.47

10 H B 0.72 -0.65 8.84 13.20 17.42 11.29 12.27

11 H B 0.72 -0.67 8.79 13.23 16.24 12.67 12.35

12 H B 0.72 -0.67 8.08 13.24 16.26 12.81 12.70

13 H B 0.74 -0.57 7.94 13.01 16.31 11.96 12.76

14 H B 0.74 -0.49 8.00 12.04 15.46 12.85 13.26

15 H B 0.73 -0.47 8.50 11.60 15.01 13.54 13.65

16 H B 0.74 -0.34 8.77 11.79 15.85 13.19 15.25

17 H E 0.74 0.04 9.01 11.31 16.63 13.33 16.53

18 C E 0.74 0.02 9.03 10.86 16.30 13.67 16.39

19 C E 0.76 0.09 9.25 10.91 14.92 13.64 15.42

20 C E 0.74 0.02 8.83 10.88 13.99 13.09 13.42

21 C E 0.74 0.04 8.08 9.74 13.03 13.00 11.98

22 C E 0.75 0.05 7.31 9.22 12.04 11.54 10.36

23 C E 0.75 0.12 6.81 9.24 11.03 10.44 9.78

24 C E 0.72 0.35 6.72 8.37 11.38 10.88 9.58

25 C E 0.71 0.26 6.37 8.95 10.87 10.51 9.48

26 C E 0.75 0.16 5.13 8.68 8.87 8.39 7.40

27 H E 0.80 0.15 5.07 8.00 9.23 7.34 8.21

28 H E 0.79 -0.03 4.73 7.41 8.34 7.10 7.02

29 H E 0.83 -0.10 4.39 7.19 8.15 6.91 6.51

30 H E 0.86 -0.13 4.36 7.05 8.61 6.87 7.74

31 H B 0.86 -0.55 4.33 6.89 8.22 6.53 7.71

32 H B 0.88 -0.59 4.13 6.65 7.74 6.50 6.46

33 H E 0.90 -0.33 4.40 7.02 8.05 6.79 7.82

34 H E 0.85 -0.23 4.52 7.13 8.53 6.76 8.71

35 H B 0.88 -0.37 4.31 7.21 7.98 6.75 7.43

36 H B 0.89 -0.21 4.28 7.63 7.87 6.69 7.48

37 H E 0.84 0.18 5.29 8.36 8.94 7.60 9.09

38 C E 0.84 0.33 5.19 8.28 9.09 7.61 8.85

39 C E 0.86 0.23 4.56 8.03 9.28 7.00 8.12

40 C B 0.90 0.05 5.02 7.97 7.98 7.24 7.17

41 H E 0.90 0.07 4.59 7.95 7.52 7.27 5.41

42 H E 0.89 -0.11 4.37 7.61 7.26 6.89 5.74

43 H B 0.90 -0.42 4.22 6.78 6.87 6.27 5.66

44 H B 0.89 -0.45 3.69 6.87 6.59 6.33 5.69

45 H E 0.90 -0.31 3.86 7.47 6.55 6.92 5.69

46 H E 0.90 -0.45 4.13 7.18 6.34 6.88 5.62

47 H B 0.90 -0.67 3.80 6.52 6.09 6.49 5.50

48 H B 0.90 -0.59 3.51 6.84 5.80 6.68 5.78

49 H E 0.90 -0.57 3.65 7.49 5.74 7.01 6.18

50 H B 0.89 -0.79 4.05 7.18 5.65 7.08 6.43

51 H B 0.88 -0.79 3.75 6.59 5.66 6.64 6.84

52 H B 0.87 -0.78 3.69 7.13 5.47 6.91 6.95

53 H B 0.86 -0.73 4.19 7.74 5.62 7.64 7.24

54 H B 0.86 -0.70 4.62 7.53 5.65 7.53 7.36

55 H E 0.87 -0.46 4.46 7.42 5.67 7.12 7.77

56 H E 0.88 -0.28 4.64 8.26 5.92 7.31 8.85

57 H B 0.88 -0.29 5.28 8.40 6.30 7.22 8.68

58 H E 0.91 -0.15 6.10 8.70 6.62 7.00 9.27

59 H E 0.90 -0.11 6.45 8.42 6.31 7.52 8.94

60 H E 0.89 -0.16 6.67 8.52 6.62 8.79 9.38

61 H B 0.90 -0.40 5.77 8.06 6.14 8.74 8.60

62 H B 0.90 -0.66 5.34 7.63 5.77 7.70 7.74

63 H E 0.91 -0.51 6.08 7.64 6.08 8.81 8.45

64 H E 0.91 -0.41 5.95 7.28 6.28 9.72 8.02

65 H B 0.91 -0.72 4.97 6.72 5.87 8.60 6.78

66 H B 0.90 -0.65 4.84 6.70 5.90 8.09 7.16

67 H E 0.91 -0.41 5.44 7.02 6.46 9.31 7.58

68 H E 0.90 -0.46 5.18 6.58 6.29 9.21 7.12

69 H B 0.92 -0.58 4.34 6.03 6.13 7.84 6.14

70 H E 0.91 -0.41 4.89 6.32 6.83 7.77 7.29

71 H E 0.90 -0.39 5.53 6.70 6.88 8.49 7.55

72 H B 0.89 -0.58 5.07 6.24 6.69 7.97 6.88

73 H B 0.90 -0.60 4.86 5.81 7.04 6.85 7.13

74 H E 0.95 -0.34 5.59 6.40 7.40 7.41 8.27

75 H E 0.94 -0.19 5.96 6.72 7.43 7.88 7.66

76 C B 0.89 -0.19 5.72 6.45 7.81 7.16 7.52

77 C B 0.82 -0.32 5.88 6.53 8.44 6.74 9.22

78 C B 0.81 -0.33 5.08 6.20 8.21 6.62 8.53

79 C E 0.85 -0.20 4.99 6.19 8.07 6.83 8.54

80 C E 0.85 -0.18 4.74 6.12 7.65 7.10 7.87

81 C E 0.88 0.02 5.05 6.61 7.78 7.24 7.39

82 C E 0.87 0.04 4.93 6.72 8.04 7.17 7.16

83 C B 0.84 -0.07 4.41 6.46 7.45 7.03 7.67

84 C E 0.79 -0.17 4.62 6.92 7.29 7.77 8.22

85 C E 0.84 0.07 4.79 7.22 8.16 7.80 8.17

86 H E 0.86 0.04 4.82 7.61 8.66 8.07 8.02

87 H E 0.89 0.13 4.70 7.62 7.97 7.07 8.38

88 H E 0.89 0.08 4.42 7.54 7.95 7.26 8.47

89 H B 0.84 -0.04 5.10 7.92 7.61 7.32 8.16

90 H B 0.85 -0.06 4.94 7.51 7.31 7.14 7.19

91 C E 0.80 0.32 5.36 8.01 7.73 8.08 8.67

92 C E 0.77 0.26 5.46 8.63 8.06 8.28 8.46

93 C E 0.81 0.22 5.52 9.13 8.24 8.48 8.58

94 C E 0.84 0.19 5.80 9.31 8.43 8.32 8.34

95 C E 0.85 0.27 6.07 9.87 8.56 8.61 9.01

96 C E 0.90 0.18 5.60 9.18 7.92 8.15 8.78

97 H E 0.91 0.01 5.71 8.93 7.82 8.89 8.75

98 H B 0.90 -0.19 6.08 9.25 8.56 9.15 8.94

99 H E 0.93 -0.06 5.39 8.33 7.68 7.96 9.38

100 H E 0.93 -0.22 4.86 7.43 6.13 7.25 8.38

101 H B 0.93 -0.39 4.71 7.25 5.89 7.05 6.86

102 H E 0.93 -0.22 4.71 7.29 5.80 6.88 6.75

103 H E 0.91 -0.10 4.21 7.38 5.94 6.54 7.22

104 H E 0.91 -0.23 3.96 6.83 5.97 6.39 6.55

105 H B 0.92 -0.40 3.95 6.60 5.99 6.43 6.02

106 H E 0.92 -0.31 3.99 7.02 6.02 6.37 6.69

107 H E 0.93 -0.24 3.76 6.60 6.24 6.02 6.60

108 H B 0.93 -0.50 3.49 6.17 6.33 6.13 5.71

109 H B 0.93 -0.56 3.57 6.83 6.24 6.28 6.34

110 H E 0.93 -0.30 3.80 7.13 6.59 6.34 6.57

111 H E 0.92 -0.41 3.75 6.75 6.91 6.16 6.16

112 H B 0.88 -0.48 3.65 6.88 6.94 6.38 6.16

113 H E 0.83 -0.14 4.04 7.89 7.29 6.61 7.08

114 H E 0.89 0.03 4.25 7.97 7.54 6.61 7.39

115 H B 0.85 -0.19 4.32 8.17 8.05 6.55 7.58

116 H B 0.79 -0.26 4.41 10.27 9.44 7.19 8.31

117 H E 0.80 -0.06 4.74 10.65 10.17 7.81 9.20

118 H E 0.80 -0.01 5.13 9.82 10.11 8.25 9.36

119 H E 0.79 -0.22 4.77 9.51 10.84 8.30 10.25

120 H E 0.77 -0.27 4.90 9.84 11.33 8.44 11.80

121 H E 0.79 -0.21 4.94 9.49 10.64 8.45 11.16

122 H B 0.81 -0.31 5.46 9.65 10.27 8.17 10.69

123 H B 0.80 -0.46 5.25 9.71 9.30 7.75 8.98

124 H B 0.81 -0.42 4.65 8.46 8.16 7.76 9.18

125 H E 0.81 -0.12 4.48 7.82 7.97 8.69 10.20

126 H E 0.79 -0.08 4.60 8.19 8.07 8.78 9.81

127 H B 0.80 -0.28 4.52 9.21 7.65 7.97 9.55

128 H B 0.78 -0.24 4.30 9.88 7.22 8.53 9.64

129 H E 0.79 0.05 4.32 10.19 7.29 9.38 9.82

130 C E 0.78 0.15 4.54 10.47 7.39 9.90 10.61

131 H E 0.77 -0.12 4.37 11.11 6.99 8.87 10.21

132 H B 0.77 -0.35 4.26 11.82 6.55 8.86 10.91

133 H E 0.79 -0.18 4.50 11.26 6.80 9.03 12.19

134 H E 0.77 -0.39 4.79 10.52 6.98 8.96 12.16

135 H B 0.76 -0.49 4.75 11.36 6.55 9.25 12.12

136 H E 0.79 -0.30 4.84 11.24 6.74 8.64 13.26

137 H E 0.79 -0.26 5.16 10.13 7.48 9.01 14.76

138 H B 0.79 -0.52 5.37 10.26 7.75 9.98 14.72

139 H E 0.79 -0.49 5.27 10.49 8.90 10.70 15.44

140 H E 0.80 -0.28 6.05 9.77 11.01 9.72 15.38

141 H E 0.79 -0.14 5.86 9.76 10.63 11.62 14.25

142 H B 0.78 -0.33 5.91 10.77 10.20 11.16 13.29

143 H E 0.79 -0.23 5.95 11.07 11.65 11.09 13.75

144 H E 0.77 -0.25 7.10 10.94 12.03 10.30 12.08

145 H E 0.79 -0.43 7.21 9.71 10.86 10.60 10.48

146 H B 0.78 -0.60 7.64 9.52 11.61 10.05 11.97

147 H B 0.79 -0.51 6.89 9.54 13.12 10.07 13.30

148 H B 0.78 -0.54 7.25 9.95 12.55 10.56 13.49

149 H B 0.77 -0.66 7.30 9.61 12.38 9.98 12.52

150 H B 0.76 -0.66 7.08 7.82 12.95 9.47 11.13

151 H B 0.75 -0.57 6.70 7.17 13.19 9.68 11.88

152 H B 0.74 -0.40 6.67 8.04 11.36 10.00 12.75

153 H B 0.74 -0.56 6.67 7.85 9.98 9.38 12.01

154 H B 0.74 -0.62 6.98 7.88 9.70 9.21 11.64

155 H E 0.74 -0.43 7.33 8.29 9.40 10.02 12.75

156 H E 0.73 -0.31 6.84 8.09 9.31 10.82 13.30

157 H B 0.71 -0.48 7.08 9.60 8.86 11.41 12.52

158 H B 0.71 -0.31 8.31 10.76 9.56 10.83 12.94

159 H E 0.69 -0.13 8.28 10.44 10.32 11.58 14.19

160 C E 0.64 -0.20 8.43 11.00 10.43 14.06 15.11

161 C B 0.63 -0.17 9.68 13.29 10.85 14.17 14.45

162 C E 0.59 0.23 10.97 14.75 12.35 14.48 15.71

163 C E 0.56 0.61 10.88 14.41 13.16 15.94 17.01

164 C E 0.50 0.89 11.72 16.31 13.35 18.20 17.85

165 C E 0.47 1.28 12.49 18.40 14.09 20.21 19.33

166 C E 0.46 1.84 13.92 20.60 15.54 22.70 21.19

**Candidate 5**

#RES SS SA COV BFP RSQ_1 RSQ_2 RSQ_3 RSQ_4 RSQ_5

1 C E 0.51 1.78 11.49 20.17 18.90 23.69 15.52

2 H E 0.62 0.84 10.61 18.16 17.50 21.97 14.26

3 H B 0.63 0.17 9.78 18.18 17.19 20.81 14.50

4 H B 0.66 -0.28 9.01 17.42 16.37 19.16 12.63

5 H B 0.66 -0.40 8.74 16.05 14.68 18.07 11.84

6 H B 0.67 -0.61 8.27 15.49 14.37 17.09 12.29

7 H B 0.67 -0.72 7.54 15.08 14.25 15.67 12.18

8 H B 0.66 -0.84 7.08 14.39 13.13 14.20 10.46

9 H B 0.67 -0.84 6.83 14.08 12.03 14.22 10.44

10 H B 0.66 -0.81 6.68 13.92 12.40 14.13 11.87

11 H B 0.68 -0.78 6.17 13.01 11.43 12.91 11.16

12 H B 0.68 -0.79 6.00 13.16 10.35 12.14 9.72

13 H B 0.69 -0.74 6.05 14.01 10.44 12.17 11.44

14 H B 0.70 -0.66 6.15 14.07 10.37 11.51 12.08

15 H B 0.71 -0.68 6.23 13.34 11.32 12.00 11.23

16 H B 0.71 -0.49 6.52 14.37 11.30 12.52 12.40

17 H E 0.71 -0.10 6.87 14.54 11.14 13.20 13.79

18 C E 0.69 -0.11 7.00 13.57 10.65 13.40 14.40

19 C E 0.66 -0.02 6.99 13.15 10.55 14.73 13.61

20 C E 0.64 0.07 6.82 13.35 10.76 15.18 12.96

21 C E 0.66 0.15 6.76 13.12 9.55 15.72 10.82

22 C E 0.66 0.19 6.46 11.90 8.96 14.98 8.17

23 C E 0.66 0.25 6.04 11.11 8.85 14.69 9.21

24 C E 0.71 0.45 5.57 10.13 8.46 13.58 8.78

25 C E 0.72 0.35 4.80 9.16 8.37 12.36 7.58

26 H E 0.76 0.18 4.25 8.28 8.00 10.90 7.52

27 H E 0.79 0.19 4.17 8.43 7.23 10.31 7.34

28 H E 0.81 -0.07 3.86 7.16 6.60 9.04 6.79

29 H B 0.83 -0.22 3.73 7.89 6.79 8.82 6.41

30 H E 0.88 -0.21 3.83 9.13 6.64 8.92 6.98

31 H B 0.88 -0.62 3.71 8.46 6.35 8.79 6.69

32 H B 0.89 -0.65 3.61 7.96 6.27 8.52 5.90

33 H E 0.91 -0.40 3.73 9.46 7.21 8.51 6.37

34 H E 0.91 -0.27 3.97 10.01 7.32 8.71 6.93

35 H B 0.90 -0.44 4.03 9.32 6.92 8.85 6.31

36 H B 0.90 -0.32 4.12 9.18 7.11 7.94 6.14

37 H E 0.89 0.13 4.28 10.57 7.54 8.04 6.85

38 C E 0.86 0.28 4.68 11.18 7.66 8.49 6.98

39 C E 0.77 0.10 5.06 10.94 8.02 8.79 7.23

40 C B 0.88 -0.04 4.92 9.95 7.99 9.27 6.59

41 H E 0.88 0.03 5.19 9.29 7.34 9.48 6.52

42 H E 0.88 -0.16 4.75 8.54 5.99 8.25 6.19

43 H B 0.91 -0.44 4.18 8.13 5.90 7.03 5.52

44 H B 0.91 -0.44 4.41 7.86 6.29 7.14 5.56

45 H E 0.89 -0.32 4.88 7.88 6.53 8.14 6.52

46 H E 0.89 -0.47 4.50 7.45 6.47 7.56 6.29

47 H B 0.91 -0.67 4.06 6.92 6.35 6.56 5.60

48 H E 0.92 -0.59 4.60 7.69 7.22 7.51 6.57

49 H E 0.91 -0.56 4.84 7.87 7.50 8.33 7.23

50 H B 0.89 -0.79 4.38 7.05 7.49 7.37 6.47

51 H B 0.89 -0.81 4.44 7.07 8.15 7.26 6.51

52 H B 0.89 -0.75 4.92 8.08 9.04 8.21 7.03

53 H B 0.87 -0.70 4.93 7.85 9.32 8.31 6.90

54 H B 0.84 -0.65 4.71 7.52 9.39 8.03 6.95

55 H E 0.86 -0.43 5.03 8.14 10.36 8.69 7.12

56 H E 0.86 -0.35 5.28 9.41 10.93 9.20 7.64

57 H E 0.86 -0.30 5.36 9.53 11.31 8.48 8.00

58 H E 0.91 -0.21 5.40 10.81 10.20 9.09 8.38

59 H E 0.94 -0.20 5.19 9.59 9.86 8.22 8.01

60 H E 0.94 -0.32 5.21 9.09 9.68 8.06 9.13

61 H B 0.92 -0.51 4.96 8.16 10.01 7.59 8.45

62 H B 0.92 -0.76 4.50 7.47 8.68 7.09 7.22

63 H E 0.95 -0.63 4.41 7.15 7.87 6.83 7.80

64 H E 0.96 -0.52 4.46 7.22 8.36 6.93 8.15

65 H B 0.95 -0.77 4.26 6.61 7.75 6.51 6.56

66 H B 0.95 -0.69 3.99 5.92 6.50 6.42 6.34

67 H E 0.96 -0.46 4.00 6.58 7.04 6.63 7.50

68 H E 0.94 -0.54 4.10 6.70 7.12 6.58 6.83

69 H B 0.94 -0.69 3.81 5.95 5.78 6.41 5.74

70 H E 0.94 -0.47 3.61 6.28 5.65 6.63 6.79

71 H E 0.91 -0.41 3.92 7.26 6.37 7.12 7.60

72 H B 0.90 -0.57 3.95 6.84 5.79 6.97 6.45

73 H B 0.89 -0.55 3.67 6.92 5.47 6.76 6.65

74 H E 0.89 -0.31 3.78 7.87 6.23 7.35 8.04

75 H E 0.87 -0.21 4.10 8.57 6.66 7.73 7.99

76 C B 0.82 -0.17 4.17 8.88 6.73 7.91 7.42

77 C B 0.75 -0.34 4.09 9.52 7.66 8.61 8.01

78 C B 0.66 -0.37 3.87 8.48 7.04 8.13 7.48

79 C E 0.71 -0.22 3.78 7.70 6.04 7.81 7.05

80 C B 0.74 -0.22 3.58 6.62 6.05 7.27 7.25

81 C E 0.76 0.01 3.77 7.11 7.32 8.06 9.51

82 C E 0.77 0.00 4.32 7.77 7.14 8.19 9.48

83 C B 0.74 -0.20 3.80 7.33 6.63 7.74 9.35

84 C E 0.74 -0.19 4.11 6.72 7.77 8.54 10.19

85 C E 0.79 0.06 4.35 7.40 8.76 9.53 13.43

86 C E 0.79 -0.08 5.00 8.77 9.71 9.52 13.98

87 C E 0.85 -0.02 5.58 8.62 9.71 9.13 15.09

88 H E 0.86 0.01 6.22 9.10 10.28 10.29 15.56

89 H E 0.87 -0.21 6.37 8.54 11.54 10.43 14.74

90 H B 0.88 -0.26 6.22 7.98 11.16 10.38 12.10

91 H E 0.88 -0.05 6.41 7.90 10.16 10.34 12.02

92 H E 0.88 0.05 5.96 7.53 10.55 10.10 11.64

93 H E 0.87 -0.10 6.61 8.06 11.64 11.17 10.10

94 H B 0.86 -0.18 6.83 8.93 10.29 12.37 8.75

95 H E 0.87 -0.16 6.32 8.64 9.35 12.92 8.44

96 H E 0.84 -0.25 5.18 6.95 10.05 10.46 7.39

97 H B 0.84 -0.56 5.19 7.56 10.90 9.52 7.23

98 H B 0.84 -0.56 5.02 7.95 9.96 8.13 7.80

99 H E 0.83 -0.38 4.83 6.89 8.72 7.56 7.31

100 H B 0.83 -0.52 4.88 7.17 7.79 9.20 7.01

101 H B 0.84 -0.63 4.63 6.66 6.36 8.50 6.38

102 H E 0.83 -0.48 4.57 6.13 7.77 8.24 6.25

103 H E 0.82 -0.38 4.59 5.94 8.50 8.42 6.11

104 H E 0.79 -0.42 4.74 6.73 8.09 9.02 6.81

105 H B 0.78 -0.53 4.38 6.21 7.73 7.91 6.04

106 H E 0.79 -0.36 4.84 6.33 8.29 7.72 5.91

107 H E 0.79 -0.30 5.10 7.39 9.00 8.60 6.76

108 H E 0.79 -0.44 4.63 6.94 8.43 8.38 7.06

109 H B 0.80 -0.61 4.27 6.29 7.90 7.15 6.30

110 H E 0.80 -0.48 4.80 7.33 9.33 8.03 7.27

111 H E 0.79 -0.34 5.04 8.32 10.30 7.82 7.80

112 H B 0.79 -0.56 4.47 7.73 9.80 7.22 7.94

113 H B 0.79 -0.66 4.52 7.69 9.56 8.00 7.90

114 H E 0.79 -0.36 5.09 8.92 11.03 7.87 8.77

115 H E 0.79 -0.28 4.90 9.28 11.59 7.72 9.49

116 H B 0.79 -0.36 4.94 8.87 10.45 8.87 9.27

117 H E 0.76 -0.15 5.44 9.92 10.74 9.83 10.01

118 H E 0.76 -0.02 5.84 10.01 12.86 10.56 10.85

119 H E 0.74 -0.10 6.51 10.70 12.63 12.85 11.30

120 H E 0.74 -0.34 6.54 10.41 10.94 12.55 11.14

121 H B 0.71 -0.51 6.53 10.21 11.13 12.91 9.96

122 H E 0.70 -0.36 7.16 10.58 12.44 13.92 10.47

123 H E 0.69 -0.48 7.67 11.45 11.71 14.50 11.15

124 H B 0.68 -0.75 7.60 10.67 11.10 13.49 10.43

125 H B 0.65 -0.76 8.16 11.82 11.75 14.51 10.16

126 H B 0.67 -0.64 8.70 12.74 12.00 15.77 11.26

127 H B 0.68 -0.65 9.15 13.33 11.91 15.68 11.85

128 H E 0.66 -0.61 9.36 13.82 13.05 15.68 11.86

129 H B 0.64 -0.28 9.52 14.07 14.22 15.46 12.73

130 C E 0.64 -0.45 9.47 13.51 13.73 15.64 12.49

131 H E 0.61 -0.51 9.23 12.34 13.35 15.48 12.24

132 H E 0.60 -0.55 8.77 10.33 13.39 14.91 11.69

133 H B 0.60 -0.57 9.19 10.70 14.50 15.16 11.23

134 H B 0.59 -0.65 9.28 10.55 14.49 15.28 11.42

135 H B 0.57 -0.68 9.39 8.96 15.33 15.36 12.17

136 H B 0.57 -0.55 10.00 9.54 16.86 15.74 13.17

137 H E 0.55 -0.24 10.83 11.20 18.06 16.56 13.49

138 H B 0.52 -0.13 11.31 11.47 18.63 15.80 14.29

139 H B 0.51 0.17 12.02 11.96 19.86 16.19 16.10

140 H E 0.53 1.12 13.05 14.16 21.59 17.99 17.40

141 C E 0.51 1.77 13.74 15.23 22.64 19.17 18.29
