# Supplementary material for: Comparative transcriptomic analysis and structure prediction of novel Newt proteins
Source: PLoS One. 2019 Aug 16;14(8):e0220416. doi: 10.1371/journal.pone.0220416 (PMC6697330; doi:10.1371/journal.pone.0220416)
Supplement: S2 Table — Based on high TM-score 10 different proteins with structural resemblance have been reported for each Newt candidate gene. PDB number, RMSD value, and percentage identity between similar proteins is also reported. (DOCX) [file pone.0220416.s002.docx]

**S2 Table. Structural resemblance of novel Newt proteins.** Based on high TM-score 10 different proteins with structural resemblance have been reported for each Newt candidate gene. PDB number, RMSD value, and percentage identity between similar proteins is also reported.

| **PDB Hit** | **Similar protein** | **Organisms** | **TM-Score** | **RMSD^a^** | **Iden^a^** |
| --- | --- | --- | --- | --- | --- |
| **Candidate 1** | | | | | |
| 2gfpb | EmrD | *E. coli* | 0.487 | 4.87 | 0.051 |
| 6cfwH | Membrane bound hydrogenase | [*Pyrococcus furiosus*](https://www.rcsb.org/pdb/search/smartSubquery.do?smartSearchSubtype=TreeEntityQuery&t=1&n=2261) | 0.482 | 4.72 | 0.058 |
| 4k82A1 | Lv-RSN-1 | *Leptodactylus vastus* | 0.482 | 2.20 | 0.152 |
| 1i6vD | RNA polymerase-rifampicin complex | [*Thermus aquaticus*](https://www.rcsb.org/pdb/search/smartSubquery.do?smartSearchSubtype=TreeEntityQuery&t=1&n=271) | 0.480 | 4.79 | 0.059 |
| [3eqlN](http://www.rcsb.org/pdb/explore/explore.do?structureId=3eql) | RNA polymerase holoenzyme | [*Thermus thermophilus*](https://www.rcsb.org/pdb/search/smartSubquery.do?smartSearchSubtype=TreeEntityQuery&t=1&n=300852) | 0.479 | 5.09 | 0.052 |
| 4fjqA | alpha-Bisabolol synthase | [*Escherichia coli*](https://www.rcsb.org/pdb/search/smartSubquery.do?smartSearchSubtype=TreeEntityQuery&t=1&n=469008) | 0.479 | 5.17 | 0.071 |
| 1xmeA | Recombinant Cytochrome ba3 Oxidase | [*Thermus thermophilus*](https://www.rcsb.org/pdb/search/smartSubquery.do?smartSearchSubtype=TreeEntityQuery&t=1&n=300852) | 0.474 | 4.68 | 0.064 |
| 4kppA | H+/Ca2+ Exchanger CAX | *Archaeoglobus fulgidus* | 0.474 | 5.15 | 0.093 |
| 3o0rB | Nitric oxide reductase | *Pseudomonas aeruginosa* | 0.474 | 4.27 | 0.031 |
| 5mz61 | Separase-Securin complex | *Caenorhabditis elegans* | 0.472 | 4.65 | 0.062 |
| **Candidate 2** | | | | | |
| 4k82A | Lv-RSN-1 | *Leptodactylus vastus* | 0.652 | 1.57 | 0.189 |
| 4dgyC | Human PARP-1 bound to a DNA double strand break | *Homo sapiens* | 0.527 | 4.43 | 0.073 |
| 1eg3A | DYSTROPHIN WW DOMAIN FRAGMENT | [*Homo*](https://www.rcsb.org/pdb/search/smartSubquery.do?smartSearchSubtype=TreeEntityQuery&t=1&n=271) *sapiens* | 0.507 | 4.65 | 0.091 |
| 5w98A | Pyridine synthase | [*Planobispora rosea*](https://www.rcsb.org/pdb/search/smartSubquery.do?smartSearchSubtype=TreeEntityQuery&t=1&n=35762) | 0.506 | 4.11 | 0.080 |
| 5uqoB | 2-methylcitrate synthase | *Aspergillus fumigatus* | 0.504 | 4.49 | 0.076 |
| 5amqA | La Crosse Bunyavirus polymerase | *Bunyavirus La Crosse* | 0.501 | 4.69 | 0.033 |
| 4tvxW | CRISPR RNA-guided surveillance complex | [*Escherichia coli*](https://www.rcsb.org/pdb/search/smartSubquery.do?smartSearchSubtype=TreeEntityQuery&t=1&n=469008) | 0.501 | 3.90 | 0.093 |
| 6cscA | Chicken citrate synthase complex | *Gallus gallus* | 0.498 | 4.37 | 0.053 |
| 2wwbA | TRANSLATING WHEAT GERM 80S RIBOSOME | [*Saccharomyces cerevisiae*](https://www.rcsb.org/pdb/search/smartSubquery.do?smartSearchSubtype=TreeEntityQuery&t=1&n=559292) | 0.497 | 4.29 | 0.056 |
| **5ehkA** | tRNA dependent lantibiotic dehydratase MibB | [*Microbispora corallina*](https://www.rcsb.org/pdb/search/smartSubquery.do?smartSearchSubtype=TreeEntityQuery&t=1&n=83302) | 0.496 | 4.41 | 0.056 |
| **Candidate 3** | | | | | |
| 4k82A | Lv-RSN-1 | *Leptodactylus vastus* | 0.567 | 3.96 | 0.851 |
| 2r7qA | Reovirus Outer-Capsid Protein mu1 | [*Reovirus type 3 (strain Dearing)*](https://www.rcsb.org/pdb/search/smartSubquery.do?smartSearchSubtype=TreeEntityQuery&t=1&n=10886)*, [Reovirus type 1 (strain Lang)](https://www.rcsb.org/pdb/search/smartSubquery.do?smartSearchSubtype=TreeEntityQuery&t=1&n=10884)* | 0.541 | 4.85 | 0.957 |
| 2cse1 | Sr-substituted LH1-RC complex | [*Thermochromatium tepidum*](https://www.rcsb.org/pdb/search/smartSubquery.do?smartSearchSubtype=TreeEntityQuery&t=1&n=1050) | 0.515 | 4.83 | 0.901 |
| 5b5mC | PHOTOSYNTHETIC REACTION CENTER | *Blastochloris viridis* | 0.507 | 4.89 | 0.872 |
| 1r2cC | lipoprotein BT2263 | *Bacteroides thetaiotaomicron* | 0.506 | 4.92 | 0.894 |
| 5fq4A | N4 mini-vRNAP P2 promoter complex | [*Enterobacteria phage N4*](https://www.rcsb.org/pdb/search/smartSubquery.do?smartSearchSubtype=TreeEntityQuery&t=1&n=10752) | 0.502 | 4.64 | 0.830 |
| 3c31A | LbaCas13a (C2c2) | *[Lachnospiraceae bacterium](https://www.rcsb.org/pdb/search/smartSubquery.do?smartSearchSubtype=TreeEntityQuery&t=1&n=1898203)* | 0.499 | 4.69 | 0.879 |
| 5w1hA | Histidine-containing Phosphotransfer Protein | [*Zea mays*](https://www.rcsb.org/pdb/search/smartSubquery.do?smartSearchSubtype=TreeEntityQuery&t=1&n=4577) | 0.498 | 4.62 | 0.808 |
| 1wn0D | phosphotransfer protein ZmHP2 | [*Zea mays*](https://www.rcsb.org/pdb/search/smartSubquery.do?smartSearchSubtype=TreeEntityQuery&t=1&n=4577) | 0.497 | 3.79 | 0.723 |
| 2q4fB | histidine-containing phosphotransfer protein | [*Oryza sativa subsp. japonica*](https://www.rcsb.org/pdb/search/smartSubquery.do?smartSearchSubtype=TreeEntityQuery&t=1&n=39947) | 0.486 | 3.96 | 0.709 |
| **Candidate 4** | | | | | |
| 4k82A | Lv-RSN-1 | *Leptodactylus vastus* | 0.656 | 2.24 | 0.188 |
| 2x79A | Inward facing conformation of Mhp1 | [*Microbacterium liquefaciens*](https://www.rcsb.org/pdb/search/smartSubquery.do?smartSearchSubtype=TreeEntityQuery&t=1&n=33918) | 0.548 | 4.20 | 0.053 |
| 3dh4D | Sodium/Sugar symporter | *vibrio parahaemolyticus* | 0.522 | 5.01 | 0.080 |
| 3I1IA | Arg-bound Escherichia coli AdiC | *Escherichia coli* | 0.511 | 5.20 | 0.038 |
| 2xq2A | K294A mutant of vSGLT | [*Vibrio parahaemolyticus*](https://www.rcsb.org/pdb/search/smartSubquery.do?smartSearchSubtype=TreeEntityQuery&t=1&n=670) | 0.507 | 4.99 | 0.080 |
| 3gj9C | ApcT Transporter Bound to 7F11 Monoclonal Fab Fragment | *[Methanocaldococcus jannaschii](https://www.rcsb.org/pdb/search/smartSubquery.do?smartSearchSubtype=TreeEntityQuery&t=1&n=243232)* | 0.505 | 4.58 | 0.046 |
| 6irsB | human LAT1-4F2hc complex | [*Homo sapiens*](https://www.rcsb.org/pdb/search/smartSubquery.do?smartSearchSubtype=TreeEntityQuery&t=1&n=9606) | 0.500 | 4.71 | 0.033 |
| 3hfxA | carnitine transporter | [*Escherichia coli (strain K12)*](https://www.rcsb.org/pdb/search/smartSubquery.do?smartSearchSubtype=TreeEntityQuery&t=1&n=83333) | 0.500 | 4.85 | 0.094 |
| 2wswA | Carnitine Transporter from Proteus mirabilis | *Proteus mirabilis* | 0.499 | 4.58 | 0.075 |
| 50qtA | cationic amino acid transporter | *[Geobacillus kaustophilus](https://www.rcsb.org/pdb/search/smartSubquery.do?smartSearchSubtype=TreeEntityQuery&t=1&n=235909)* | 0.498 | 5.02 | 0.070 |
| Candidate 5 | | | | | |
| 4k82A | Lv-RSN-1 | *Leptodactylus vastus* | 0.640 | 2.52 | 0.162 |
| 4dgyC | Human PARP-1 bound to a DNA double strand break | *Homo sapiens* | 0.513 | 4.21 | 0.042 |
| 6c75A | Iron containing alcohol dehydrogenase | *Thermococcus thioreducens* | 0.510 | 4.66 | 0.054 |
| 5x3xQ | cobalt energy-coupling factor transporter-CbiMQO | [*Rhodobacter capsulatus*](https://www.rcsb.org/pdb/search/smartSubquery.do?smartSearchSubtype=TreeEntityQuery&t=1&n=272942) | 0.508 | 4.14 | 0.077 |
| 5yvmA | archaeal halo-thermophilic Red Sea brine pool alcohol dehydrogenase ADH/D1 bound to NZQ | [*candidate division MSBL1 archaeon SCGC-AAA259E19*](https://www.rcsb.org/pdb/search/smartSubquery.do?smartSearchSubtype=TreeEntityQuery&t=1&n=1698264) | 0.509 | 4.89 | 0.023 |
| 3iv7B | Iron-containing alcohol dehydrogenase | *[Corynebacterium glutamicum](https://www.rcsb.org/pdb/search/smartSubquery.do?smartSearchSubtype=TreeEntityQuery&t=1&n=196627)* | 0.506 | 4.58 | 0.063 |
| 1eg3A | Dystrophin ww domain fragment in complex with a beta-dystroglycan peptide | [*Homo sapiens*](https://www.rcsb.org/pdb/search/smartSubquery.do?smartSearchSubtype=TreeEntityQuery&t=1&n=9606) | 0.502 | 4.55 | 0.130 |
| 3o1qA | Helicobacter pylori Urease Accessory Protein UreF | *Helicobacter pylori* | 0.501 | 4.21 | 0.069 |
| 2bi4B | 1,2-propanediol oxidoreductase | [*Escherichia coli*](https://www.rcsb.org/pdb/search/smartSubquery.do?smartSearchSubtype=TreeEntityQuery&t=1&n=562) | 0.501 | 4.50 | 0.053 |
| 3bfjM | 1,3-propanediol oxidoreductase | *[Klebsiella pneumoniae](https://www.rcsb.org/pdb/search/smartSubquery.do?smartSearchSubtype=TreeEntityQuery&t=1&n=573)* | 0.500 | 4.60 | 0.047 |
