# Supplementary material for: Comparative transcriptomic analysis and structure prediction of novel Newt proteins
Source: PLoS One. 2019 Aug 16;14(8):e0220416. doi: 10.1371/journal.pone.0220416 (PMC6697330; doi:10.1371/journal.pone.0220416)
Supplement: S3 Table — First two PDB hits, Ligands, C-score, and Ligand binding site residue for all 5 novel Newt proteins have been listed. (DOCX) [file pone.0220416.s003.docx]

**S3 Table. Predicted ligand, and ligand binding site of Newt proteins.** First two PDB hits, Ligands, C-score, and Ligand binding site residue for all 5 novel Newt proteins have been listed

| **Candidate 1** | | | |
| --- | --- | --- | --- |
| PDB Hit | Ligand Name | C-score | Ligand binding residue |
| 1sofF | HEME B | 0.10 | 90,94,113,120,121 |
| 1vd5A | Glycine residue | 0.07 | 94, 113 |
| **Candidate 2** | | | |
| 4bpdA | (78M) (2S)-2,3-DIHYDROXYPROPYL(7Z)-PENTADEC-7-ENOATE | 0.08 | 40,43,44,47,48,61,62,65,84,87 |
| 4n3eC | (2AN) 8-ANILINO-1-NAPHTHALENE SULFONATE | 0.05 | 79,83 |
| **Candidate 3** | | | |
| 2r1rA | (Peptide) | 0.12 | 94,95,96,97,98,99,102 |
| 2rh1A | (CLR) CHOLESTEROL | 0.05 | 71, 90 |
| **Candidate 4** | | | |
| 4m70A | Peptide | 0.05 | 43,46,72,76 |
| 4us4A | (78M) (2S)-2,3-DIHYDROXYPROPYL(7Z)-PENTADEC-7-ENOATE | 0.05 | 73,74,78,83 |
| **Candidate 5** | | | |
| 2dysN | (DCW) 1,3-DICYCLOHEXYLUREA | 0.08 | 47, 73 |
| 5d56A | (78M) (2S)-2,3-DIHYDROXYPROPYL(7Z)-PENTADEC-7-ENOATE | 0.07 | 29,32,33,36,50,51,66,69 |
