# Supplementary material for: Comparative transcriptomic analysis and structure prediction of novel Newt proteins
Source: PLoS One. 2019 Aug 16;14(8):e0220416. doi: 10.1371/journal.pone.0220416 (PMC6697330; doi:10.1371/journal.pone.0220416)
Supplement: S4 Table — First two consensus prediction of GO terms based on molecular function, biological process, and cellular component for the corresponding Newt candidate proteins have been listed. (DOCX) [file pone.0220416.s004.docx]

**S4 Table.** **Prediction of enriched GO terms.** First two consensus prediction of GO terms based on molecular function, biological process, and cellular component for the corresponding Newt candidate proteins have been listed.

| **Candidate 1** | | | |
| --- | --- | --- | --- |
|  | **Category** | **GO Number** | **GO score** |
| **Molecular Function** | electron acceptor and electron donor in an electron transport chain | **GO: 0009055** | **0.14** |
|  | Interacting selectively and non-covalently with heme, any compound of iron complexed in a porphyrin (tetrapyrrole) ring. | **GO: 0022900** | **0.14** |
| **Biological Process** | A process in which a series of electron carriers operate together to transfer electrons from donors to any of several different terminal electron acceptors to generate a transmembrane electrochemical gradient. | **GO: 0022900** | **0.14** |
|  | The enzymatic release of energy from inorganic and organic compounds (especially carbohydrates and fats) which requires oxygen as the terminal electron acceptor. | **GO: OOO9060** | **0.14** |
| **Cellular Component** | The membrane surrounding a cell that separates the cell from its external environment. It consists of a phospholipid bilayer and associated proteins. | **GO: 0005886** | **0.14** |
|  | The protein complexes that form the electron transport system (the respiratory chain), associated with a cell membrane | **GO: 0070469** | **0.14** |
| **Candidate 2** | | | |
| **Molecular Function** | metalloprotease activity | **GO: 0008237** | **0.10** |
|  | Catalysis of the hydrolysis of N-terminal dipeptides from a polypeptide chain. | **GO: 0008239** | **0.10** |
| **Biological process** | Any process that localizes a substance or cellular component. This may occur via movement, tethering or selective degradation. | **GO: 0051234** | **0.37** |
| **Cellular component** | Part of a peptide sequence or some other covalently attached group such as a GPI anchor, which spans or is embedded in one or both leaflets of the membrane. | **GO: 0031224** | **0.37** |
| **Candidate 3** | | | |
| **Molecular Function** | Conveys a signal across a cell to trigger a change in cell function or state. | **GO:0004871** | **0.14** |
|  | Electron acceptor and electron donor in an electron transport chain | **GO:0000160** | **0.07** |
| **Biological process** | Phosphorelay signal transduction system | **GO:0000160** | **0.14** |
|  | Photolysis | **GO:0019684** | **0.07** |
| **Cellular Component** | The membrane surrounding a cell that separates the cell from its external environment. It consists of a phospholipid bilayer and associated proteins. | **GO: 0005886** | **0.07** |
|  | A plasma membrane protein-pigment complex that may be closely or peripherally associated to photosynthetic reaction centers that participate in harvesting and transferring radiant energy to the reaction center. | **GO: 0030077** | **0.07** |
| **Candidate 4** | | | |
| **Molecular Function** | Enables the active transport of a solute across a membrane by a mechanism whereby two or more species are transported in opposite directions in a tightly coupled process not directly linked to a form of energy other than chemiosmotic energy. | **GO:0015297** | **0.15** |
|  | Catalysis of the reaction: donor + hydrogen peroxide = oxidized donor + 2 H2O. | **GO:0004601** | **0.14** |
| **Biological Process** | A metabolic process that results in the removal or addition of one or more electrons to or from a substance, with or without the concomitant removal or addition of a proton or protons. | **G0: 0055114** | **0.14** |
|  | The directed movement of carnitine into, out of or within a cell, or between cells, by means of some agent such as a transporter or pore. | **GO:0015879** | **0.07** |
| **Cellular component** | The membrane surrounding a cell that separates the cell from its external environment. It consists of a phospholipid bilayer and associated proteins. | **GO: 0005886** | **0.15** |
|  | The component of a membrane consisting of the gene products and protein complexes having at least some part of their peptide sequence embedded in the hydrophobic region of the membrane. | **GO: 0016021** | **0.15** |
| **Candidate 5** | | | |
| **Molecular Function** | Catalysis of an oxidation-reduction (redox) reaction | **GO:0016491** | **0.37** |
|  | Interacting selectively and non-covalently with any metal ion. | **GO:0046872** | **0.37** |
| **Biological Process** | A metabolic process that results in the removal or addition of one or more electrons to or from a substance, with or without the concomitant removal or addition of a proton or protons. | **GO:0055114** | **0.31** |
| **Cellular Component** | All of the contents of a cell excluding the plasma membrane and nucleus, but including other subcellular structures. | **GO: 0005737** | **0.12** |
